# A Controller Synthesis Method to Achieve Independent Reference Tracking Performance and Disturbance Rejection Performance

**DOI:** 10.1021/acsomega.2c01524

**Published:** 2022-04-29

**Authors:** Gengjin Shi, Shaojie Liu, Donghai Li, Yanjun Ding, YangQuan Chen

**Affiliations:** † State Key Lab of Power Systems, Department of Energy and Power Engineering, Tsinghua University, Beijing 100084, China; ‡ Mechatronics, Embedded Systems and Automation (MESA) Lab, School of Engineering, University of California, Merced, California 95343, United States

## Abstract

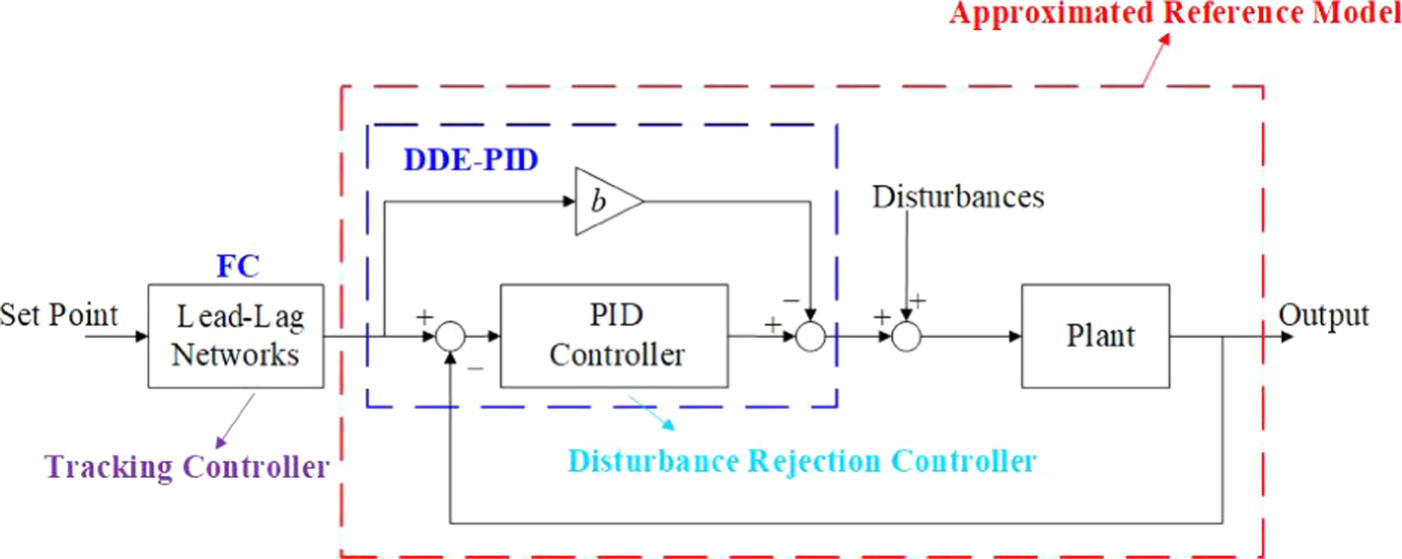

This paper deals with the conflict between the input–output response and the disturbance–output response, which cannot be completely eliminated by traditional and advanced control strategies without using the accurate process model. The inherently close association of these two responses and the unavailability of the accurate process model pose a great challenge to field test engineers of a coal-fired power plant, that is, the design requirements of reference tracking and disturbance rejection are compromised. In this paper, a novel two-degree-of-freedom controller—feedforward compensated (FC) desired dynamic equational (DDE) proportional–integral–derivative (PID) (FC-DDE PID)—is proposed as a viable alternative. In addition to achieving independent reference tracking performance and disturbance rejection performance, its simple structure and tuning procedure are specifically appealing to practitioners. Simulations, experiments, and field tests demonstrate the advantages of the proposed controller in both reference tracking and disturbance rejection, thus making FC-DDE PID a convenient and effective controller for the control of the coal-fired power plants, readily implementable on the distributed control system (DCS).

## 1 Introduction

Coal-fired power plants are still dominating the global power supply.^1^ In 2020, coal-fired power generation occupied 65% of the total power generation in China, even though renewable energies such as wind and solar have grown vigorously in the recent decade. Due to the continuous increase of power demand and the randomness of renewable energies, an increasing number of coal-fired units participate in the deep peak-shaving by regulating their output power frequently according to the automatic generation control (AGC) command. To guarantee the operating efficiency of the coal-fired power plants, all control loops should respond to the AGC command as soon as possible, which requires a faster tracking performance of controllers. Moreover, various disturbances and strong couplings between feedback control loops may affect the safety of the unit, which means that controllers should have strong ability to suppress disturbances. Generally, for the control design of a coal-fired power plant, tracking performance and disturbance rejection performance are both of significance.

Nowadays, proportional−integral−derivative (PID) controller^2^ is remaining as the first choice for engineers in thermal engineering because of its simple structure and reliable control performance. According to a survey conducted in more than 100 boiler–turbine units in Guangdong Province, China, a single-loop PI/PID controller is applied to 98.1% of feedback loops in power plants.^3^ As is known to all, the regular PI/PID-based control strategies have one-degree-of-freedom (1-DOF) structure, which means that only one controller can be designed and tuned in the closed-loop system. As a result, in the classical feedback system, feedback acts not only to modify the influence of disturbances but also to determine the reference tracking response, which leads to the compromise between control requirements.^4^ Moreover, advanced control strategies such as model predictive control (MPC),^5,6^ sliding mode control (SMC),^7,8^ and robust control^9^ are also proposed based on the 1-DOF structure so that their reference–output response and disturbance–output response are conflicting.

To overcome the shortcoming of the classical 1-DOF control system, in 1955, a new approach to feedback control—conditional feedback (CF)—was proposed by Lang et al,^4^ but it was not defined as a two-degree-of-freedom (2-DOF) control strategy at that time. The concept of 2-DOF control was first proposed and applied to the design of the PI/PID controller by Horowitz in 1963.^10^ With the development of the control technology, 2-DOF-based control systems are generally divided into two types: “feedback and feedforward” and “feedback and disturbance observer (DOB).”^11^ The former one mainly consists of the 2-DOF PI/PID controller,^12−14^ whose tuning rules have been studied by worldwide researchers, such as maximum sensitivity (*M*_s_)-constrained integral gain optimization (MIGO),^15^ internal model control (IMC),^16,17^ relative delay method,^18^ desired dynamic equational (DDE) method,^19^ multi-objective optimization,^20^ and so forth. The latter one mainly focuses on the design of the observer to handle disturbances and uncertainties, including DOB,^21^ perturbation observer (POB),^22^ equivalent-input-disturbance (EID),^23^ uncertainty and disturbance estimator (UDE),^24^ generalized PI observer (GPIO),^25^ unknown input observer (UIO),^26^ extended state observer (ESO),^27,28^ and so forth. The aforementioned 2-DOF control strategies can largely eliminate conflicts between reference–output response and disturbance–output response, which have been demonstrated by simulations and experiments. However, their applications to the control of power plants are limited for the following reasons:

(1) CF and most of the DOB-based strategies are designed based on the accurate model of the process. However, for thermal processes in the power plant, their accurate models are difficult to obtain.
(2) In terms of 2-DOF PI/PID and DOB-based strategies, they are unable to eliminate the conflict between reference tracking and disturbance rejection completely.

Above all, as for coal-fired power plants, there is an urgent need to provide a simple and practical control strategy which can not only achieve independent tracking performance and disturbance rejection performance but also has little dependency on the accurate process model. Based on this motivation and keeping simplicity into account, this paper proposes a feedforward compensated DDE PID (FC-DDE PID) controller. The main contributions of this paper are as follows:

(1) An FC-DDE PID controller is proposed to separate the input–output response and the disturbance–output response completely without using the process model.
(2) The step-by-step tuning procedure of the proposed controller is summarized.
(3) The advantages of the FC-DDE PID are demonstrated by several simulation examples and an experiment on a water tank.
(4) The FC-DDE PID is tested in a practical coal-fired power plant, and field test results show its potential.

The rest of this paper is organized as follows: The problem formulation is introduced in Section 2, followed by the design and the tuning procedure of FC-DDE PID in Section 3 and Section 4, respectively. In Section 5, the effectiveness of the proposed controller is demonstrated by several simulation examples. Moreover, in Section 6, an experiment on the water tank illustrates the merits of FC-DDE PID in both reference tracking and disturbance rejection6. Particularly, our proposed new method is demonstrated by field tests. Finally, concluding remarks are presented in the last section.

## 2 Problem Formulation

According to Section 1, the control design of coal-fired power plants should satisfy the following requirements:

(1) For a thermal process of a coal-fired power plant, only its input and output are available for the tuning or design of the controller, so an accurate process model should be unnecessary for the controller design.
(2) Both tracking performance and disturbance rejection are of importance. As a result, the controller should have the ability to eliminate the conflict between the input–output response and the disturbance–output response completely.

However, based on the analyses in Appendix A, four typical control systems, that can be applied to the control of power plants, are unable to satisfy these requirements, which are presented in Table 1.

**Table 1:** Characteristics of Different Control Systems

type	typical strategies	eliminate the conflict	necessity of accurate process models
1-DOF	PID, MPC, SMC	none	some strategies are necessary
2-DOF	2-DOF PID	partially	some strategies are necessary
DOB-based	DOB, POB, UDE	partially	some strategies are necessary
CF	CF	completely	necessary

Note that the conflict specifically refers to the conflict between the input–output response and the disturbance–output response in this paper. For some strategies, the accurate process model is necessary for the controller design, which is discussed as follows. For example, as for the 1-DOF control strategy, MPC must be designed based on the accurate model of the process and it will obtain poor performance when the model is mismatched.^29^ Moreover, DOB and UDE should be designed based on the model of the nominal system.^30,31^ Some 2-DOF PID design methods, such as using the pole search technique,^32^ are developed based on the differential equations of the process. In terms of CF, its tracking controller is designed based on the inverse process model.^33^

From Table 1, it is obvious that the listed control systems are unable to eliminate the conflict completely without using accurate process models. Therefore, this paper aims at proposing a controller that can achieve independent reference tracking performance and disturbance rejection performance without using the accurate process model for the control of coal-fired power plants.

## 3 A Controller Synthesis Method—Feedforward Compensated DDE PID

**Figure 1:**
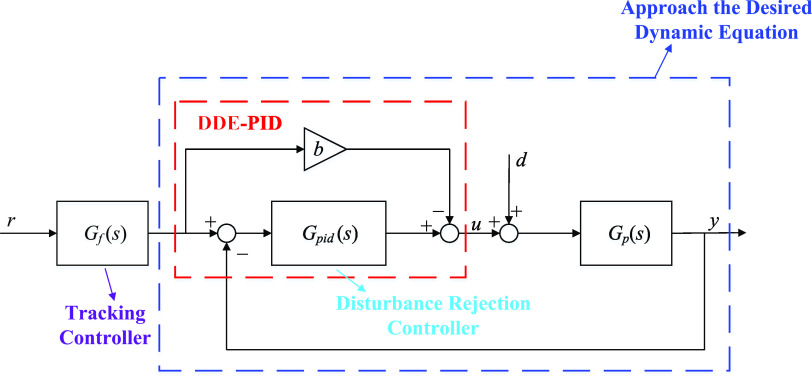
Structure of the FC-DDE PID.

In this section, we design an FC-DDE PID to solve the aforementioned problems of typical control systems. Figure 1 illustrates the structure of FC-DDE PID.

In this paper, *r*, *u*, *d*, and *y* are the set point, the control signal, the disturbance, and the output, respectively. Besides, *G*_p_(*s*) represents the transfer function of the plant and *G*_PID_(*s*) represents that of the PID controller. In terms of FC-DDE PID, *G*_f_(*s*) is the feedforward compensation, which is designed as the tracking controller.

First, we briefly introduce the DDE PI/PID. The derivations of its principles are detailed in Appendix B. The desired dynamic equations, known as the reference models of DDE PI/PID, are depicted as
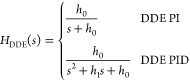
1where *H*_DDE_(*s*) denotes the transfer functions of desired dynamic equations of DDE PI/PID. In Expression (1), *h*_0_ and *h*_1_ are defined as the coefficients of *H*_DDE_(*s*). Then the parameters of DDE PI/PID are given as
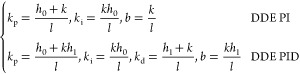
2where *k*_p_, *k*_i_, and *k*_d_ are known as the proportional, integral, and derivative gains of the PID controller while *b* refers to the feedforward coefficient of DDE PI/PID. Moreover, let
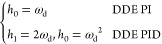
3where ω_d_ is defined as the closed-loop desired bandwidth. Therefore, tunable parameters of DDE PI/PID are *k*, *l*, and ω_d_. Note that if the process has a pure time delay, *H*_DDE_(*s*) should be selected as Expression (1) with the time delay.^34^

Second, we focus on the design of *G*_f_(*s*). The feedforward compensation is designed as the tracking controller, which is in the following form:
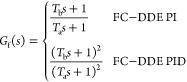
4where *T*_a_ and *T*_b_ denote the tunable parameters of the tracking controller, and *T*_a_ is usually smaller than *T*_b_ for a faster tracking response. Based on Figure 1, the transfer functions from *r* and *d* to *y* can be depicted as
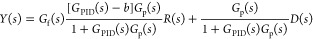
5If DDE PI/PID is tuned well, its closed-loop output can track the response of the reference model precisely, which means that
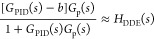
6Let *T*_b_ = 1/ω_d_; based on eq 6,eq 5 can be rewritten as
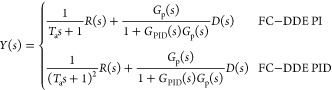
7According to eq 7, it is obvious that the input–output response is only modified by the tracking controller while the disturbance–output response is only determined by the PI/PID controller. Therefore, the FC-DDE PI/PID can eliminate the conflict without using the accurate process model, but the premise is that DDE PI/PID should be well-tuned.

## 4 Tuning Procedure of FC-DDE PID

In this section, the tuning procedure of the proposed controller is summarized. The reference tracking performance and the disturbance rejection performance should be tuned separately.

First, we focus on the design of *G*_f_(*s*), which determines the reference tracking performance of FC-DDE PI/PID. According to Section 3, *T*_b_ should be set as 1/ω_d_, while *T*_a_ should be set based on tracking requirements, such as the desired settling time *T*_sd_. For example, based on ±2% criterion, *T*_a_ should be selected as 4*T*_sd_ and 8*T*_sd_ for FC-DDE PI and FC-DDE PID, respectively.

**Figure 2:**
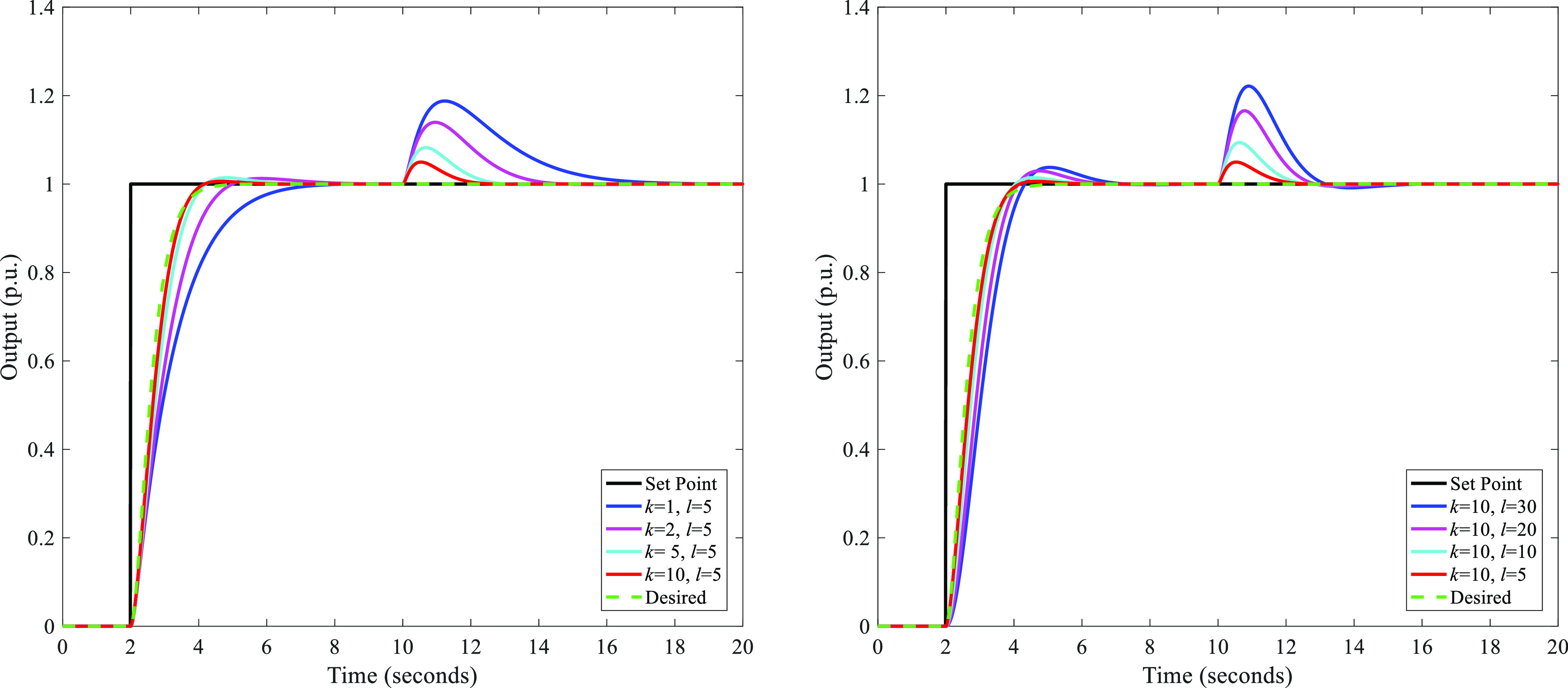
Influence of *k* and *l* on the control performance of DDE PID.

Second, we focus on the tuning of DDE PI/PID, which determines the disturbance rejection performance of the proposed controller. Since ω_d_ is chosen based on the reference model of DDE PI/PID, the tunable parameters are *k* and *l*. Figure 2 shows the influence of *k* and *l* on the control performance of DDE PID. Note that a simple plant in the form of 1/[(*s* + 1)(0.2*s* + 1)] is taken as the example, and ω_d_ is equal to 3.

From Figure 2, we can conclude that a larger *k* and a smaller *l* mean a stronger disturbance rejection and a closer output to the desired response. According to Appendix B, we can learn that *k* is equivalent to the gain of the TC disturbance observer. For a better control performance, *k* is recommended to be given as 3–10ω_d_. In this paper, *k* is given as 10ω_d_.

Moreover, it can be learned from Appendix B that *l* is related to the gain of the general system. Actually, *l* determines the sign of the control action. For example, if the process has a positive gain, *l* should be set in the range of (0, +∞).

**Figure 3:**
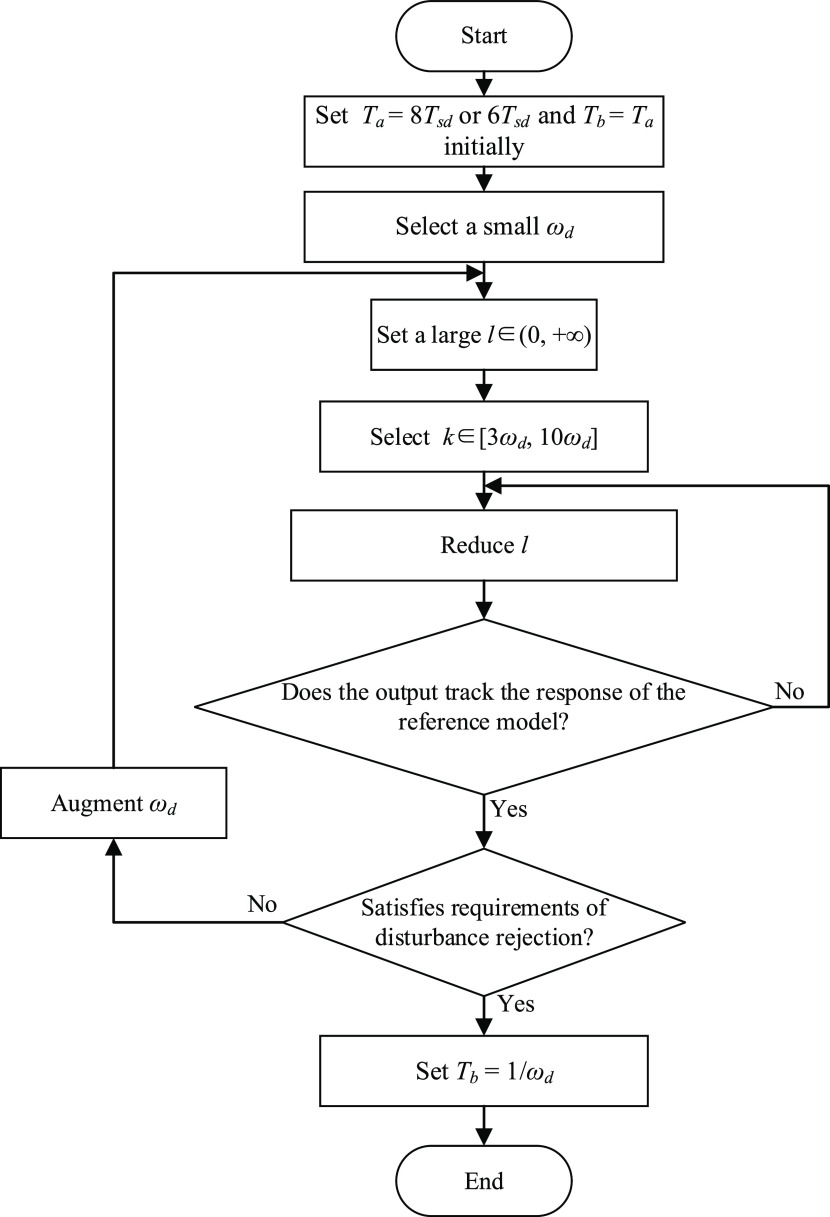
Flow chart of the tuning procedure of FC-DDE PI/PID.

Based on the aforementioned analyses, a step-by-step tuning procedure for the proposed controller is summarized as a flow chart in Figure 3. Note that the plant with a negative gain has a similar tuning procedure with the difference of *l* ∈ (−∞, 0).

## 5 Illustrative Examples

In this section, ten typical processes are selected as plants for numerical simulations to demonstrate the effectiveness of FC-DDE PI/PID. Transfer function models of these typical processes are presented in Table 2. They can describe almost all types of industrial processes, such as thermal processes and chemical processes.

**Table 2:** Transfer Function Models of Ten Typical Processes

process	type	transfer function model
*G*_p1_(*s*)	low-order process	
*G*_p2_(*s*)	high-order process	
*G*_p3_(*s*)		
*G*_p4_(*s*)		
*G*_p5_(*s*)	dead-time process	
*G*_p6_(*s*)		
*G*_p7_(*s*)	non-minimum phase process	
*G*_p8_(*s*)	integral process	
*G*_p9_(*s*)		
*G*_p10_(*s*)	unstable process	

### 5.1 Separation of Two Responses

**Figure 4:**
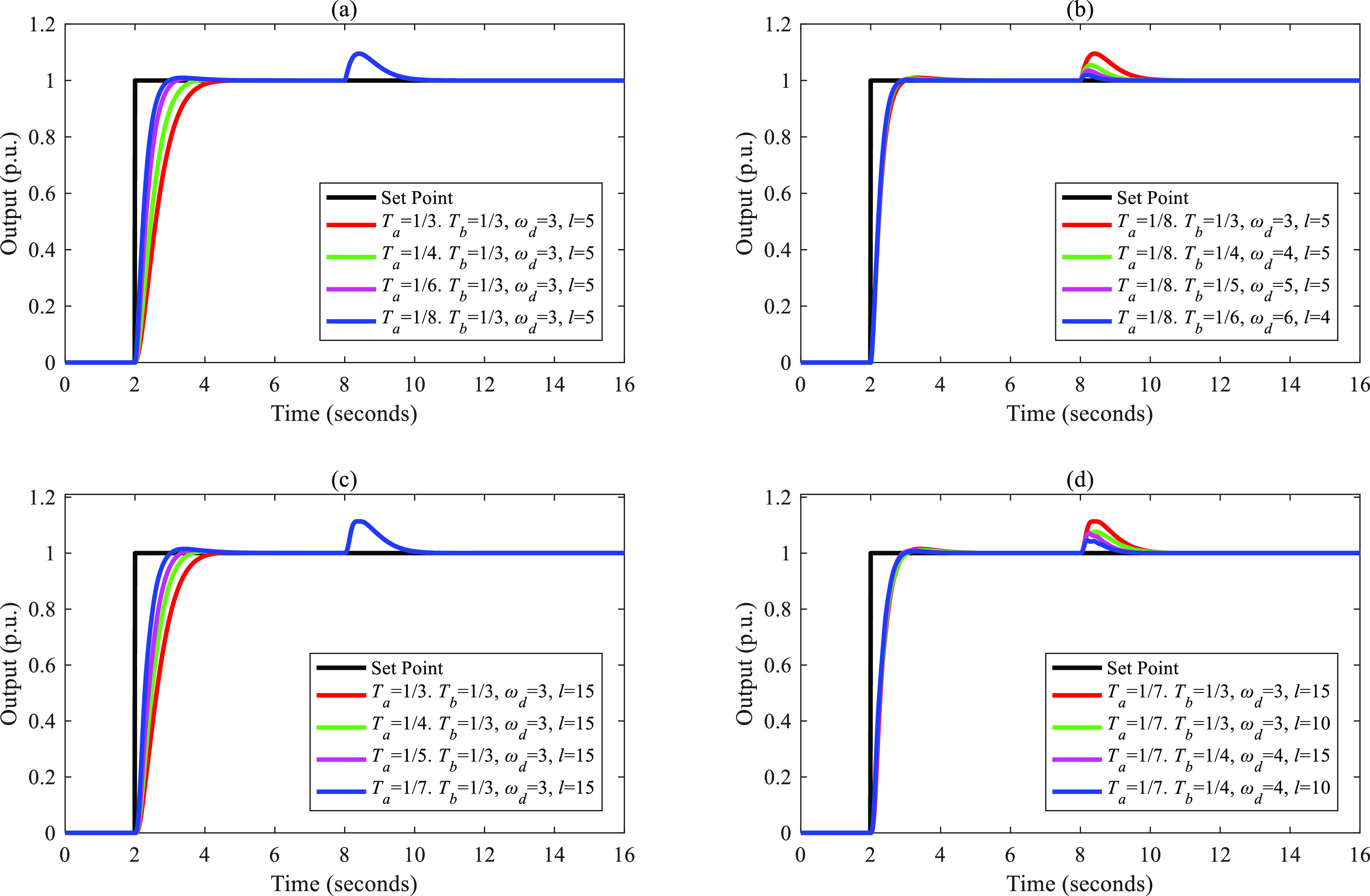
Separations of the input–output response and the disturbance–output response of FC-DDE PID: (a, b) *G*_p1_; (c, d) *G*_p2_.

**Figure 5:**
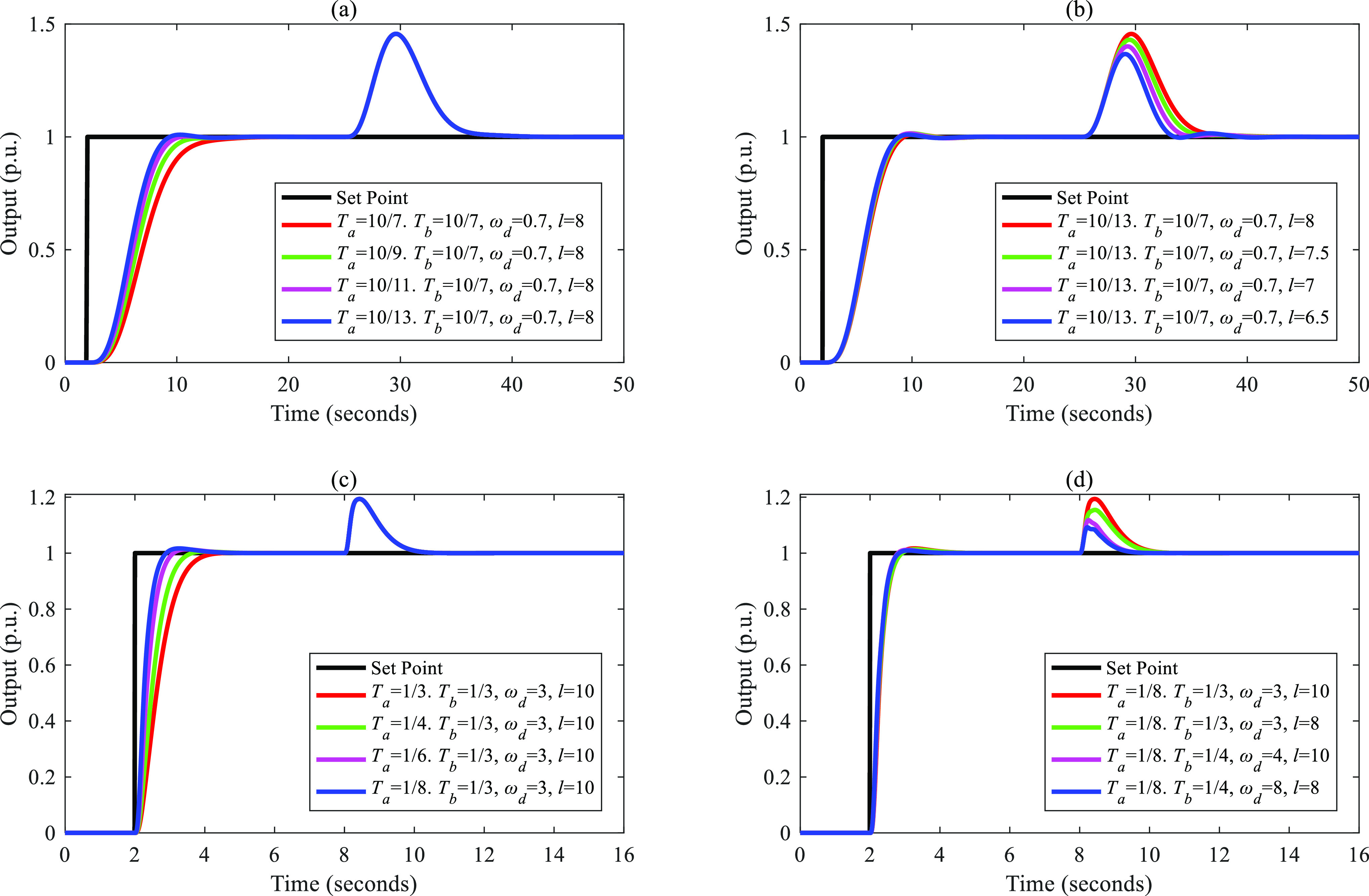
Separations of the input–output response and the disturbance–output response of FC-DDE PID: (a, b) *G*_p3_; (c, d) *G*_p4_.

**Figure 6:**
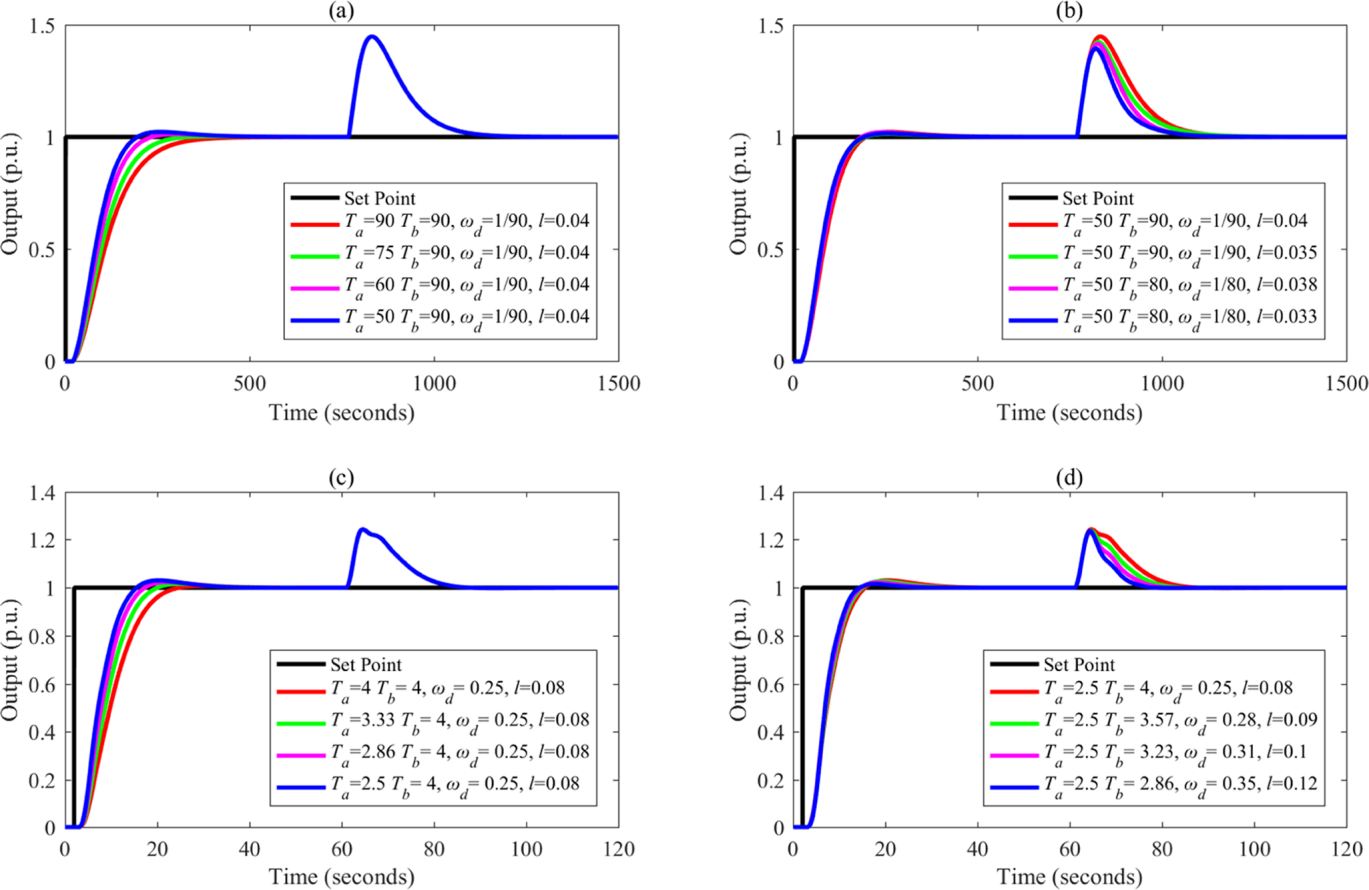
Separations of the input–output response and the disturbance–output response of FC-DDE PID: (a, b) *G*_p5_; (c, d) *G*_p6_.

**Figure 7:**
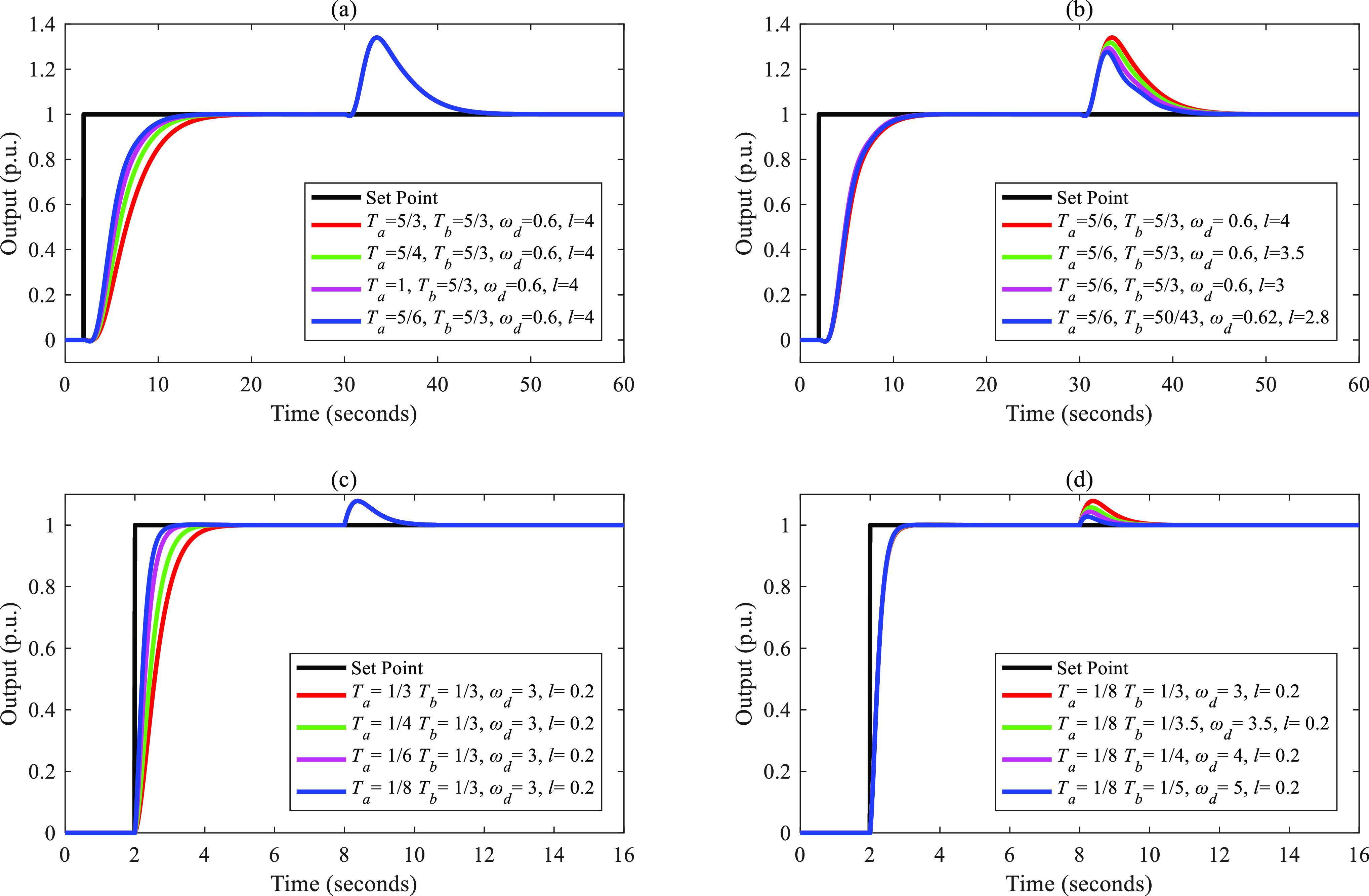
Separations of the input–output response and the disturbance–output response of FC-DDE PID: (a, b) *G*_p7_; (c, d) *G*_p8_.

**Figure 8:**
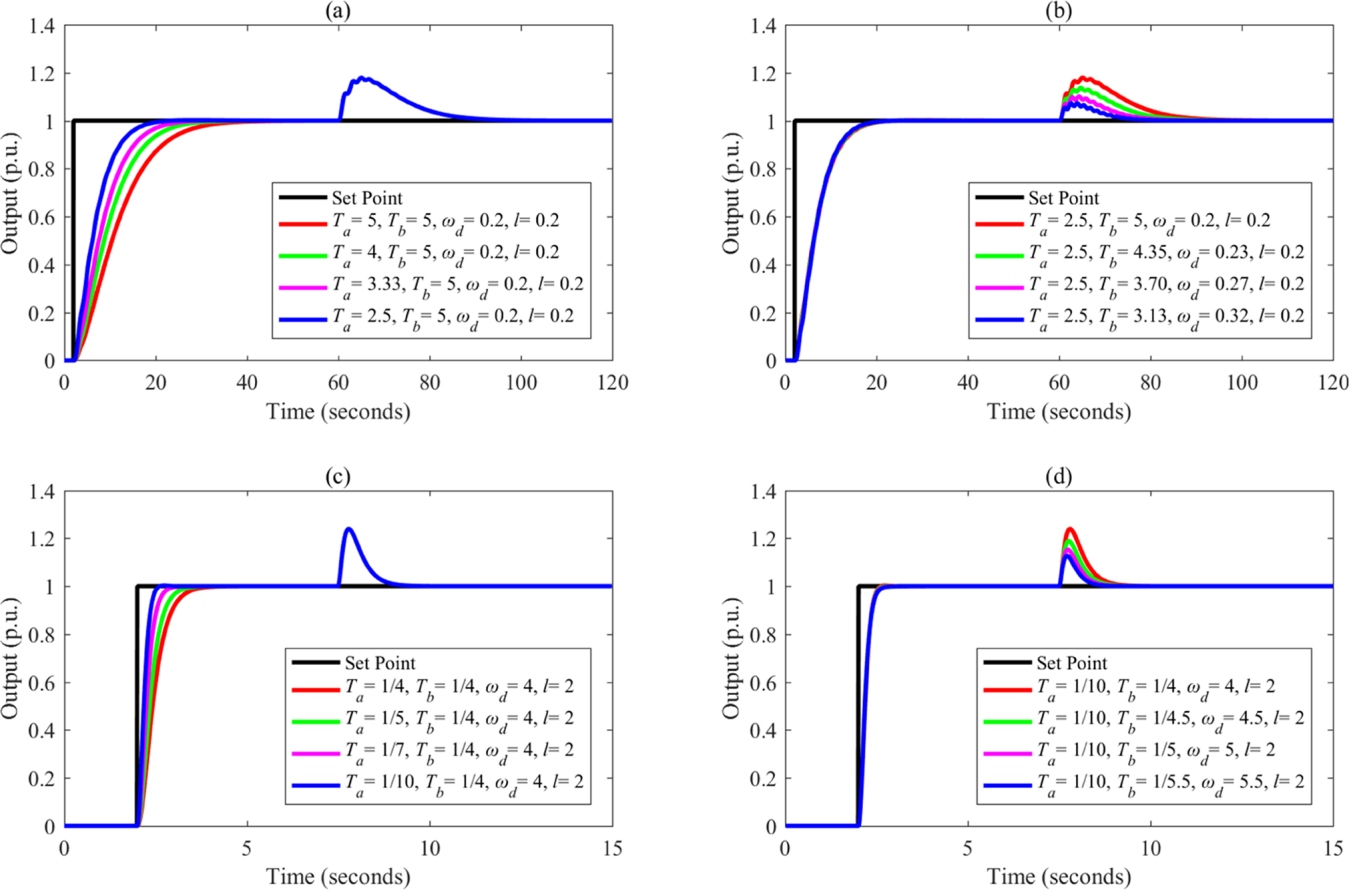
Separations of the input–output response and the disturbance–output response of FC-DDE PID: (a, b) *G*_p9_; (c, d) *G*_p10_.

In this subsection, the separation of the input–output response and the disturbance–output response is demonstrated by numerical simulations. Note that the reference tracking performance is being modified based on fixed *T*_b_ and parameters of DDE PI/PID, while the disturbance rejection performance is being tuned based on a fixed *T*_a_. Figures 4–8 show the simulation results.

From Figures 4–8, it is obvious that the disturbance–output response remains unchanged when *T*_a_ is augmented, while the input–output responses are almost fixed when *T*_b_ and parameters of DDE PID are modified. Based on the above simulations, we can conclude that the proposed controller can achieve independent reference tracking performance and disturbance rejection performance without using the accurate process model.

### 5.2 Comparisons with Other PID Controllers

To demonstrate the merits of the proposed FC-DDE PI/PID in reference tracking and disturbance rejection, PID controllers based on the Skogestad IMC (SIMC) method,^35,36^ approximated MIGO (AMIGO) method,^37^ and conventional DDE method^19,38^ are selected as comparative controllers. SIMC and AMIGO are simple tuning methods that offer highly effective quantitative calculations, so they are regarded as better choices for PID tuning in engineering.^39^Table 3 lists the parameters of different controllers. Note that AMIGO PID has the same structure as DDE PID, as shown in Figure 1. Besides, *K*_p_, *T*_i_, and *T*_d_ represent the proportional gain, the integral time, and the derivative time of SIMC PID and AMIGO PID, respectively.

**Table 3:** Parameters of Different Controllers^a^

*G*_p_(*s*)	PI/PID	SIMC {*K*_p_, *T*_i_, *T*_d_}	AMIGO {*K*_p_, *T*_i_, *T*_d_, *b*}	DDE {*l*, *k*, ω_d_}	FC-DDE {*l*, *k*, ω_d_, *T*_a_}
*G*_p1_(*s*)	PID	{5, 0.8, 0.1}	{5.15, 0.4381, 0.0487, 5.15}	{5, 30, 3}	{4, 60, 6, 1/8}
*G*_p2_(*s*)	PID	{6.67, 0.4, 0.15}	{2.2333, 0.5294, 0.0719, 2.2333}	{15, 30, 3}	{10, 40, 4, 1/7}
*G*_p3_(*s*)	PID	{0.5, 1.5, 1}	{0.47, 2.0755, 0.8333, 0.47}	{8, 7, 0.7}	{6.5, 7, 0.7, 10/13}
*G*_p4_(*s*)	PID	{17.9, 0.224, 0.22}	{3.5446, 0.5388, 0.0711, 3.5446}	{10, 30, 3}	{8, 80, 8, 1/8}
*G*_p5_(*s*)	PI	{4, 160, 0}	{2.1599, 106.6407, 0, 2.1599}	{0.04, 1/9, 1/90}	{0.033, 1/8, 1/80, 50}
*G*_p6_(*s*)	PID	{10, 8, 2}	{4.925, 8.5854, 0.9722, 4.925}	{0.08, 2.5, 0.25}	{0.12, 3.5, 0.35, 2.5}
*G*_p7_(*s*)	PID	{1.3, 2, 1.2}	{0.9653. 2.2118, 0.6248, 0.9653}	{4, 6, 0.6}	{2.8, 6.2, 0.62, 5/6}
*G*_p8_(*s*)	PID	{1.4, 2.86, 1.33}	{0.45, 13.52, 0.0845, 0}	{0.2, 30, 3}	{0.2, 50, 5, 1/8}
*G*_p9_(*s*)	PID	{0.0625, 8, 8}	—a	{0.2, 2, 0.2}	{0.2, 3.2, 0.32, 2.5}
*G*_p10_(*s*)	PID	{8.9286, 0.8, 0.8}	—a	{2, 40, 4}	{2, 55, 5.5, 1/10}

a Note: the AMIGO PID is inapplicable for *G*_p9_(*s*) and *G*_p10_(*s*).

**Figure 9:**
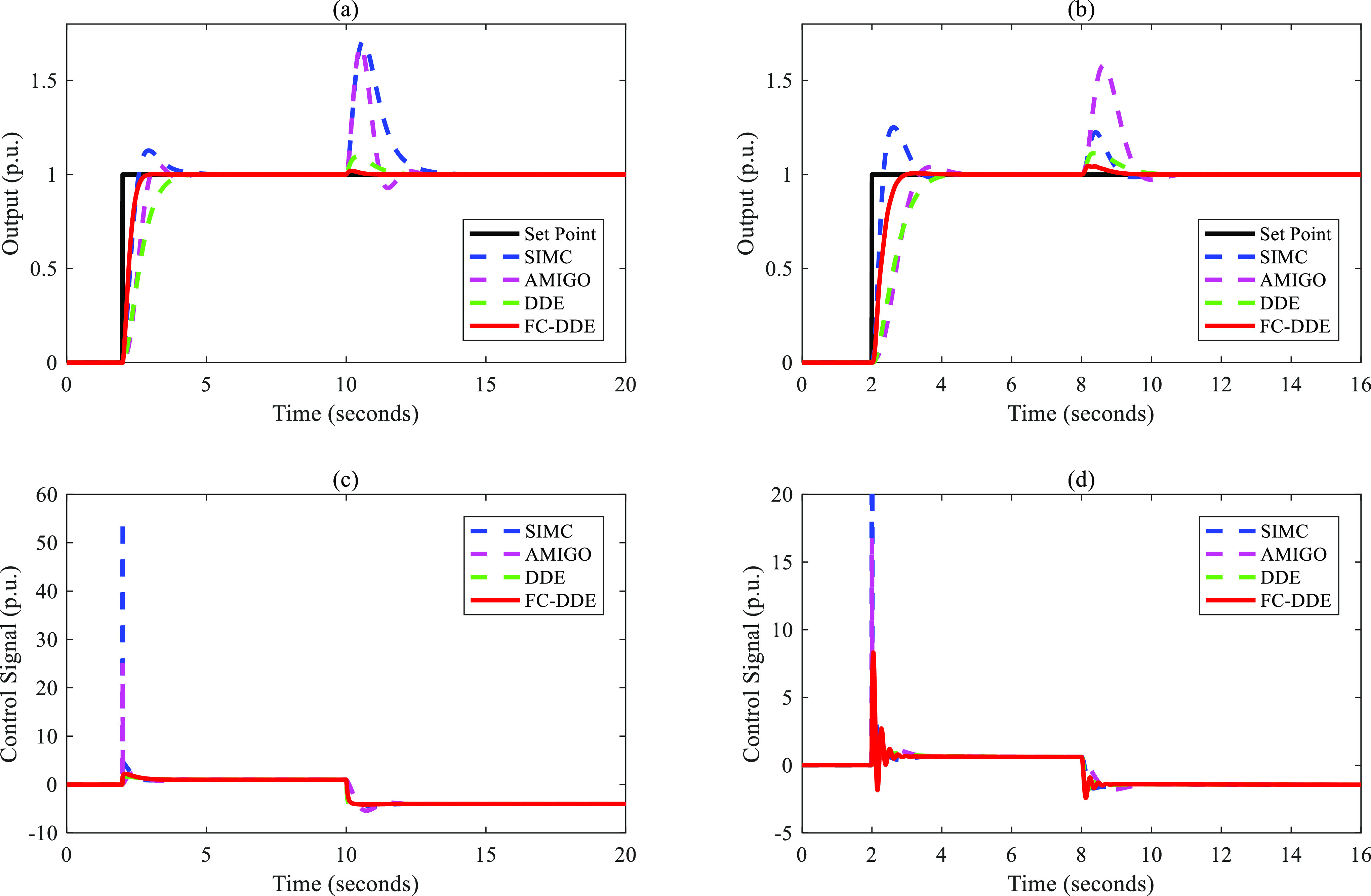
Control performance of different controllers: (a, c) *G*_p1_; (b, d) *G*_p2_.

**Figure 10:**
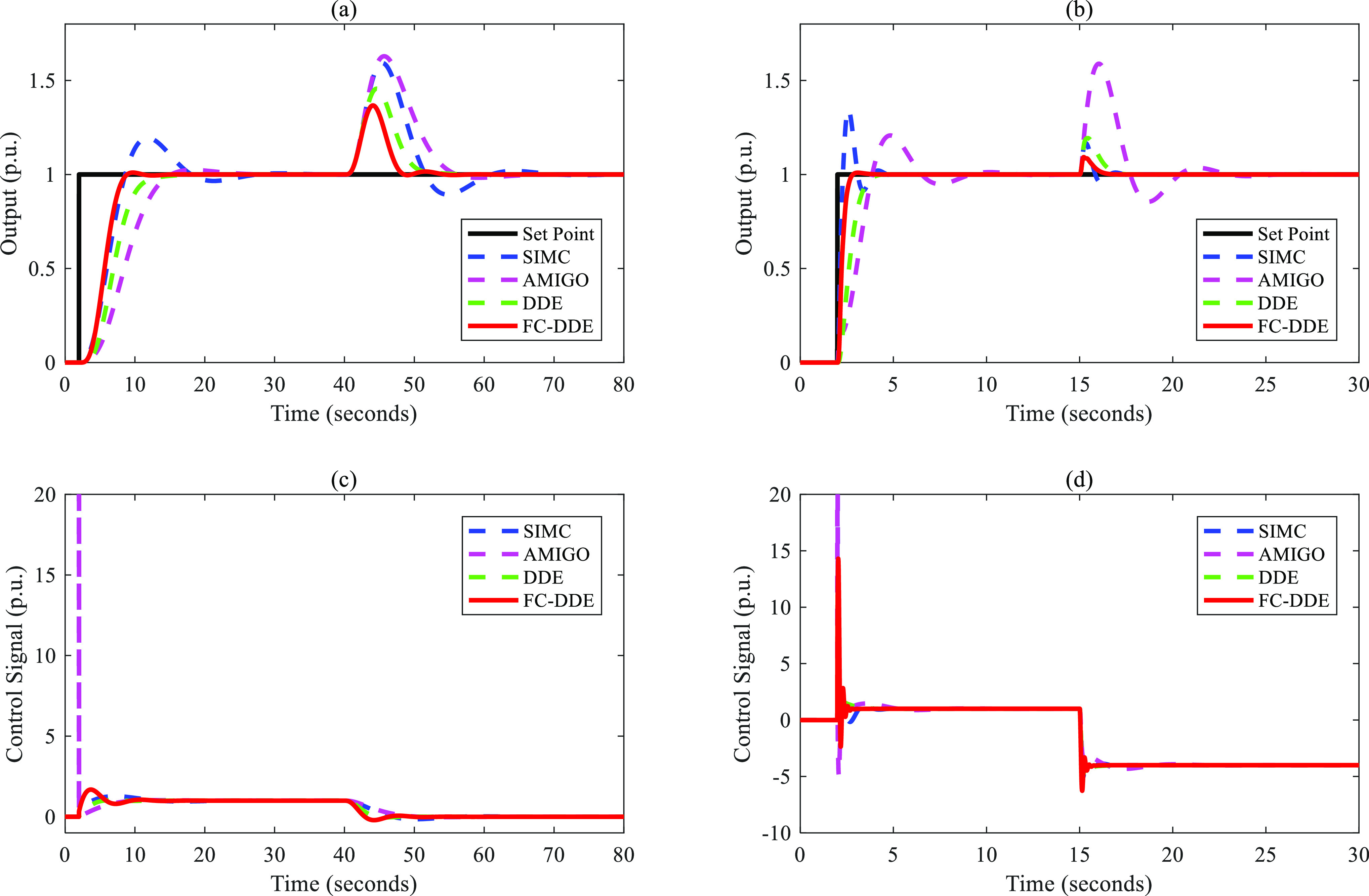
Control performance of different controllers: (a, c) *G*_p3_; (b, d) *G*_p4_.

**Figure 11:**
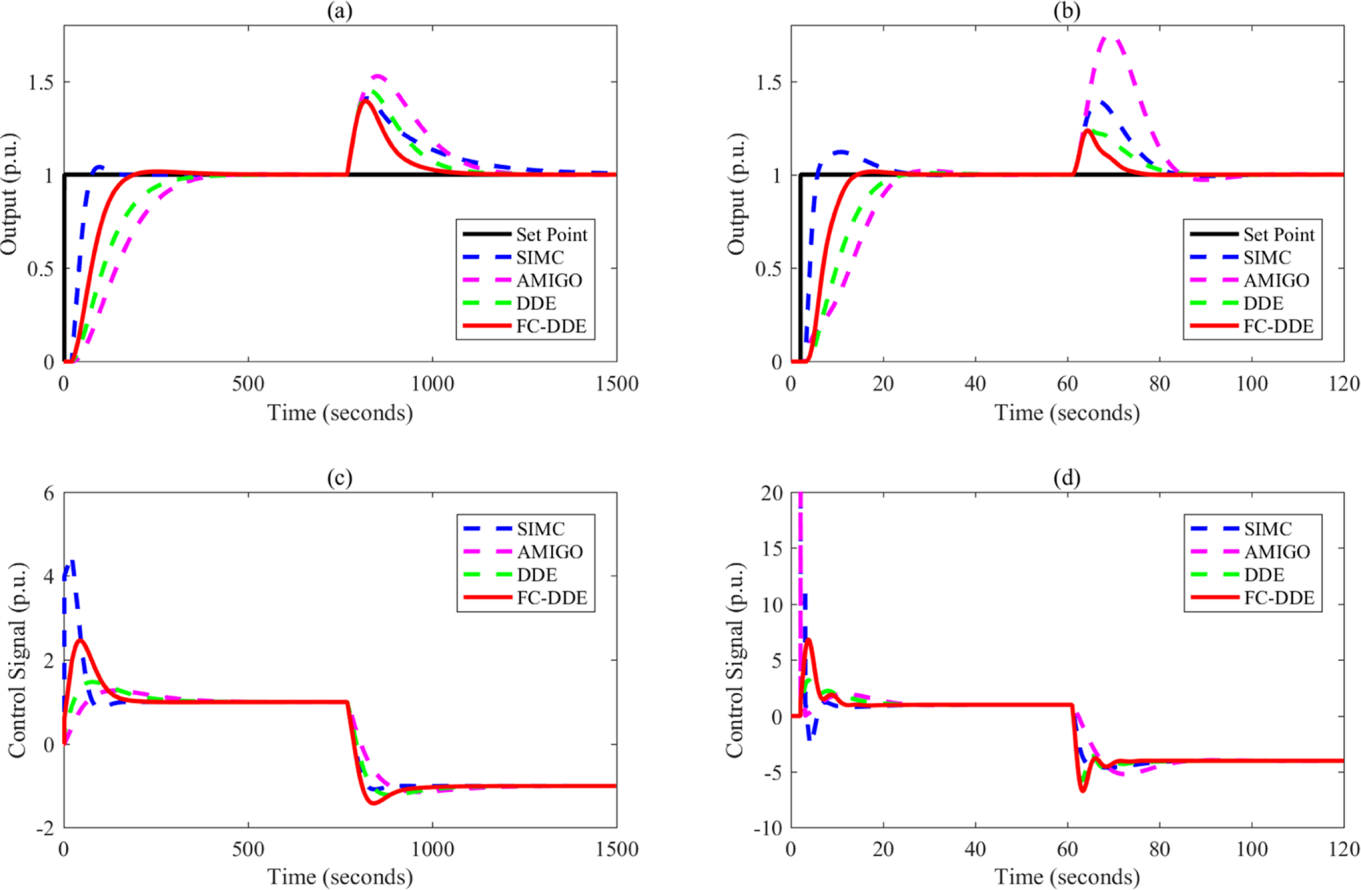
Control performance of different controllers: (a, c) *G*_p5_; (b, d) *G*_p6_.

**Figure 12:**
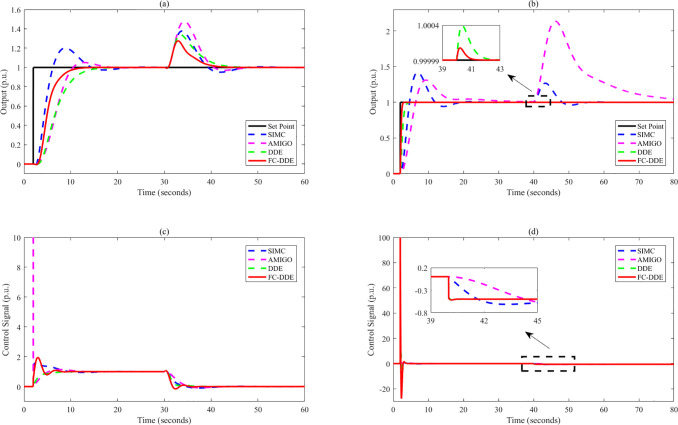
Control performance of different controllers: (a, c) *G*_p7_; (b, d) *G*_p8_.

**Figure 13:**
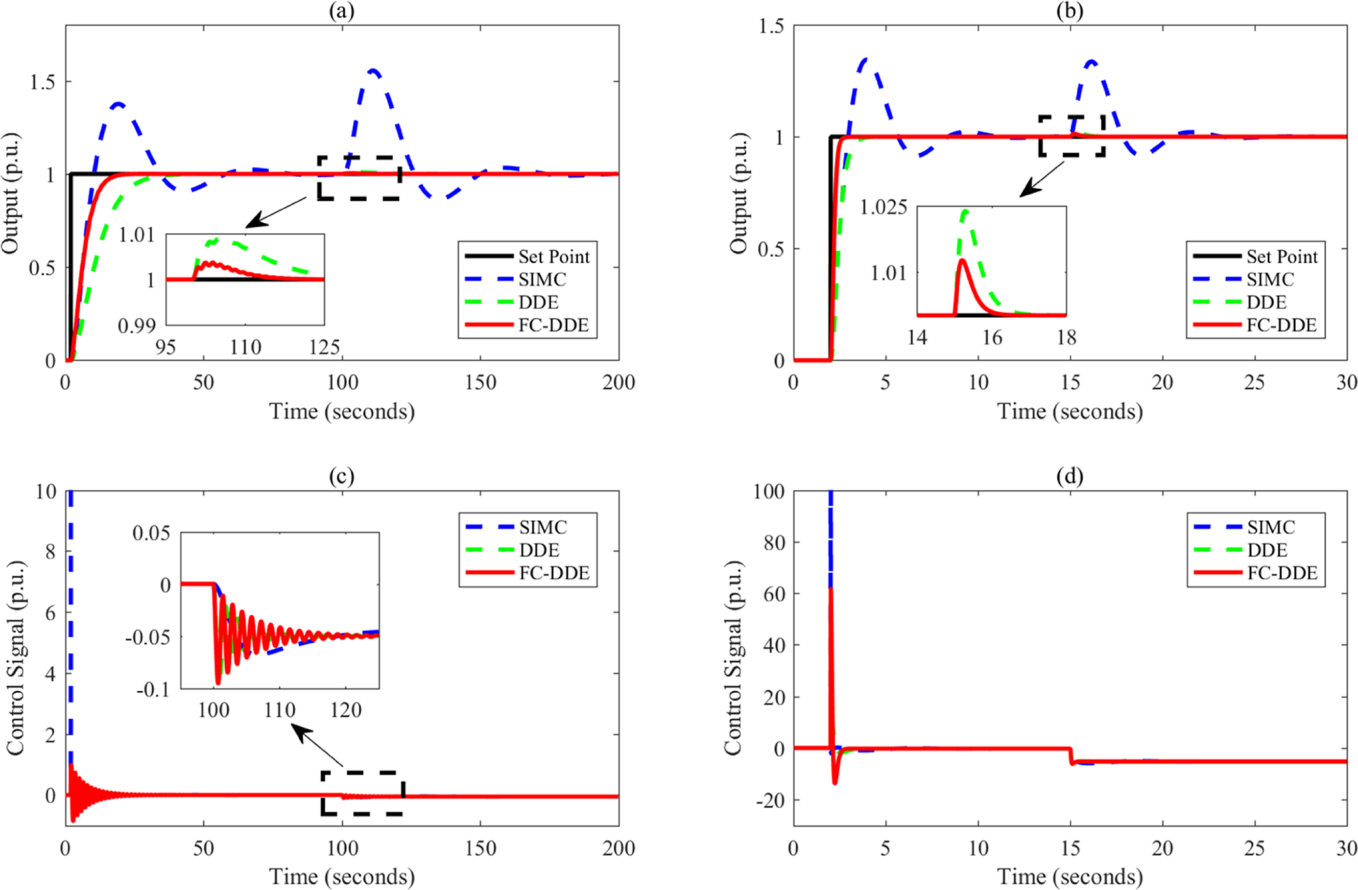
Control performance of different controllers: (a, c) *G*_p9_; (b, d) *G*_p10_.

Based on the parameters listed in Table 3, the control performance of different controllers is illustrated in Figures 9–13. Note that the step point has a unit step change at 2 s, and a step disturbance is added during the simulation.

From Figures 9–13, the following facts are obvious:

(1) Compared with SIMC PID and AMIGO PID, DDE PID and the proposed controller have more moderate tracking performance and better disturbance rejection performance.
(2) The tracking performance and disturbance rejection performance can be largely improved if DDE PID is modified as FC-DDE PID.

To evaluate the control performance quantitatively, Table 3 lists dynamic indices of different controllers, including the overshoot σ, the settling time *T*_s_, the integral absolute error (IAE), and the travel variation (TV) of the control signal. Note that IAE_sp_ is defined as the IAE of reference tracking, while IAE_ud_ is defined as that of disturbance rejection. Besides, the TV is evaluated as ∑_k=1_^∞^|*u*_k+1_–*u*_k_| (Table 4).

**Table 4:** Dynamic Indices of Different Controllers for All Processes

*G*_p_(*s*)	controller	σ (%)	*T*_s_ (s)	IAE_sp_	IAE_ud_	TV
*G*_p1_(*s*)	SIMC	12.75	2.01	0.3918	0.8000	114.99
	AMIGO	5.56	1.57	0.5742	0.4954	62.78
	DDE	0	1.88	0.6858	0.0927	8.06
	FC-DDE	0.16	0.71	0.2534	0.0093	8.66
*G*_p2_(*s*)	SIMC	25.07	1.33	0.3465	0.1389	56.06
	AMIGO	4.02	2.15	0.7362	0.5013	48.18
	DDE	0.18	1.83	0.7066	0.1138	5.79
	FC-DDE	0.67	0.82	0.3059	0.0320	34.36
*G*_p3_(*s*)	SIMC	19.46	22.29	5.2400	4.2254	33.03
	AMIGO	2.41	18.24	4.6451	6.8104	22.45
	DDE	0	10.62	5.1901	2.3328	2.18
	FC-DDE	1.01	6.41	3.7330	1.4756	4.54
*G*_p4_(*s*)	SIMC	34.47	2.26	0.3651	0.1047	84.48
	AMIGO	20.75	6.60	1.4011	1.2571	61.27
	DDE	0.10	1.83	0.7056	0.1856	10.59
	FC-DDE	0.94	0.70	0.2754	0.0625	52.55
*G*_p5_(*s*)	SIMC	4.05	121.11	443.3693	79.0546	10.45
	AMIGO	0.19	366.31	156.6926	99.1053	3.83
	DDE	0	324.56	122.3987	64.7978	4.39
	FC-DDE	1.58	169.32	81.9490	42.2402	6.77
*G*_p6_(*s*)	SIMC	12.04	20.06	3.4475	4.1612	103.81
	AMIGO	1.99	21.43	10.6995	9.2455	69.26
	DDE	0.72	19.72	8.6748	2.5966	16.63
	FC-DDE	1.54	11.17	5.5076	1.4183	25.69
*G*_p7_(*s*)	SIMC	20.16	16.92	3.4937	2.0162	35.58
	AMIGO	5.15	14.16	4.9729	2.5688	23.11
	DDE	0	12.56	5.1864	1.8587	2.12
	FC-DDE	0	8.66	3.3963	1.1819	5.25
*G*_p8_(*s*)	SIMC	36.35	15.26	3.1306	1.3614	376.33
	AMIGO	31.06	28.28	4.9692	14.6131	9.50
	DDE	0	1.94	0.6668	0.0004	104.33
	FC-DDE	0	0.74	0.2513	0.0001	706.93
*G*_p9_(*s*)	SIMC	37.61	68.59	11.5523	10.5630	20.26
	DDE	0	28.90	10.0003	0.1250	1.83
	FC-DDE	0	14.48	5.0062	0.0305	15.91
*G*_p10_(*s*)	SIMC	34.41	7.64	1.1759	0.7397	219.70
	DDE	0	1.45	0.4992	0.0156	32.29
	FC-DDE	0	0.55	0.1997	0.0060	156.29

According to Table 3, compared with SMIC PID, AMIGO PID, and DDE PID, the proposed controller has the shortest settling time and the smallest IAE_sp_ and IAE_ud_ for most processes, which shows that FC-DDE PID is superior in both reference tracking and disturbance rejection. Moreover, the overshoot of FC-DDE PID is acceptable, though it is larger than that of DDE PID. However, for the first-order plus dead time (FOPDT) system depicted as *G*_p5_(*s*), it seems that SIMC PID has better tracking performance than the proposed controller.

Additionally, as for most processes, the TV of FC-DDE PID is usually larger than that of DDE PID and smaller than those of SIMC PID and AMIGO PID, except for G_p8_(*s*). In terms of the integral process, it is obvious that FC-DDE PID may lead to the severe oscillation of the control signal.

**Figure 14:**
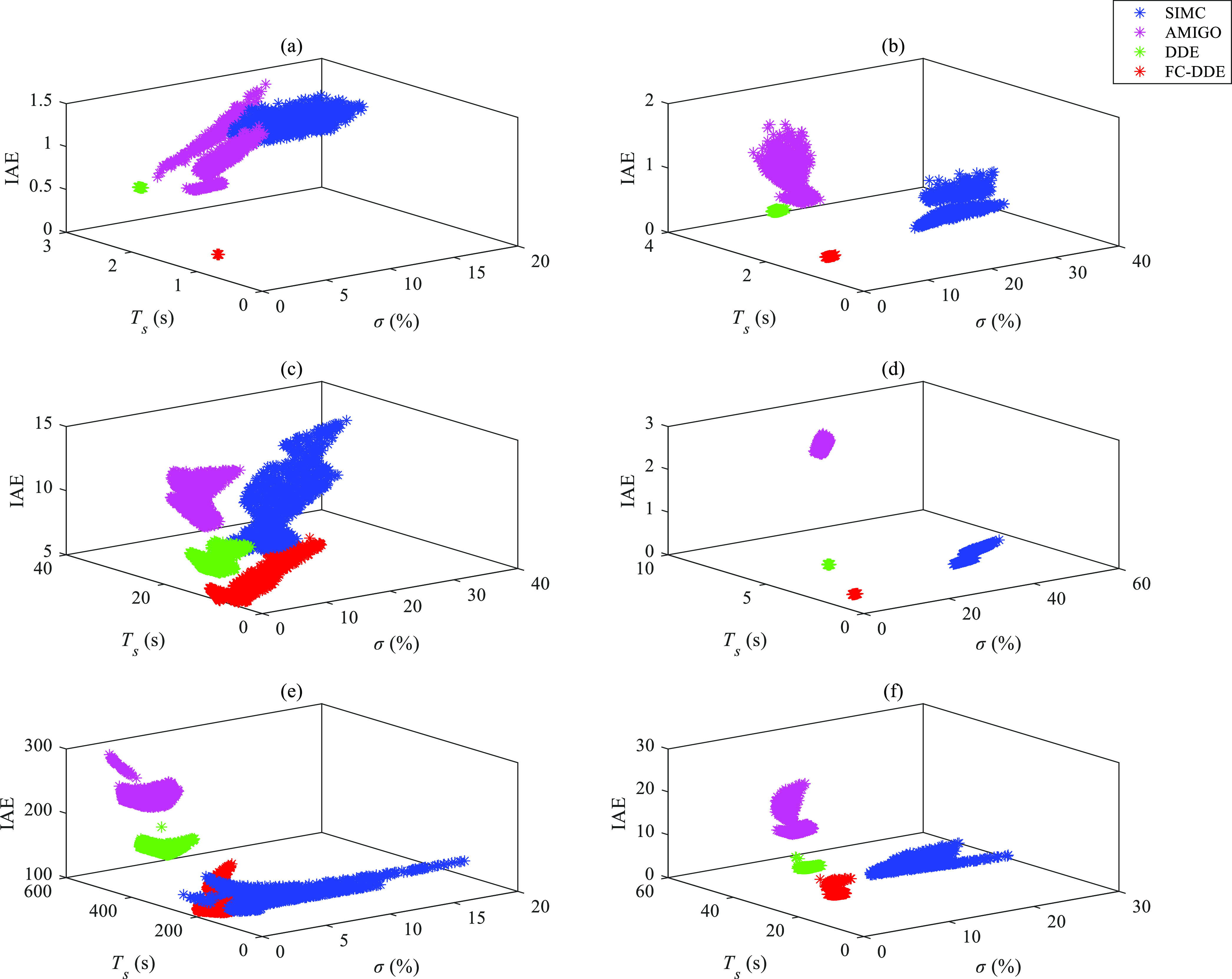
Results of Monte Carlo trials for each process: (a) *G*_p1_; (b) *G*_p2_; (c) *G*_p3_; (d) *G*_p4_; (e) *G*_p5_; and (f) *G*_p6_.

**Figure 15:**
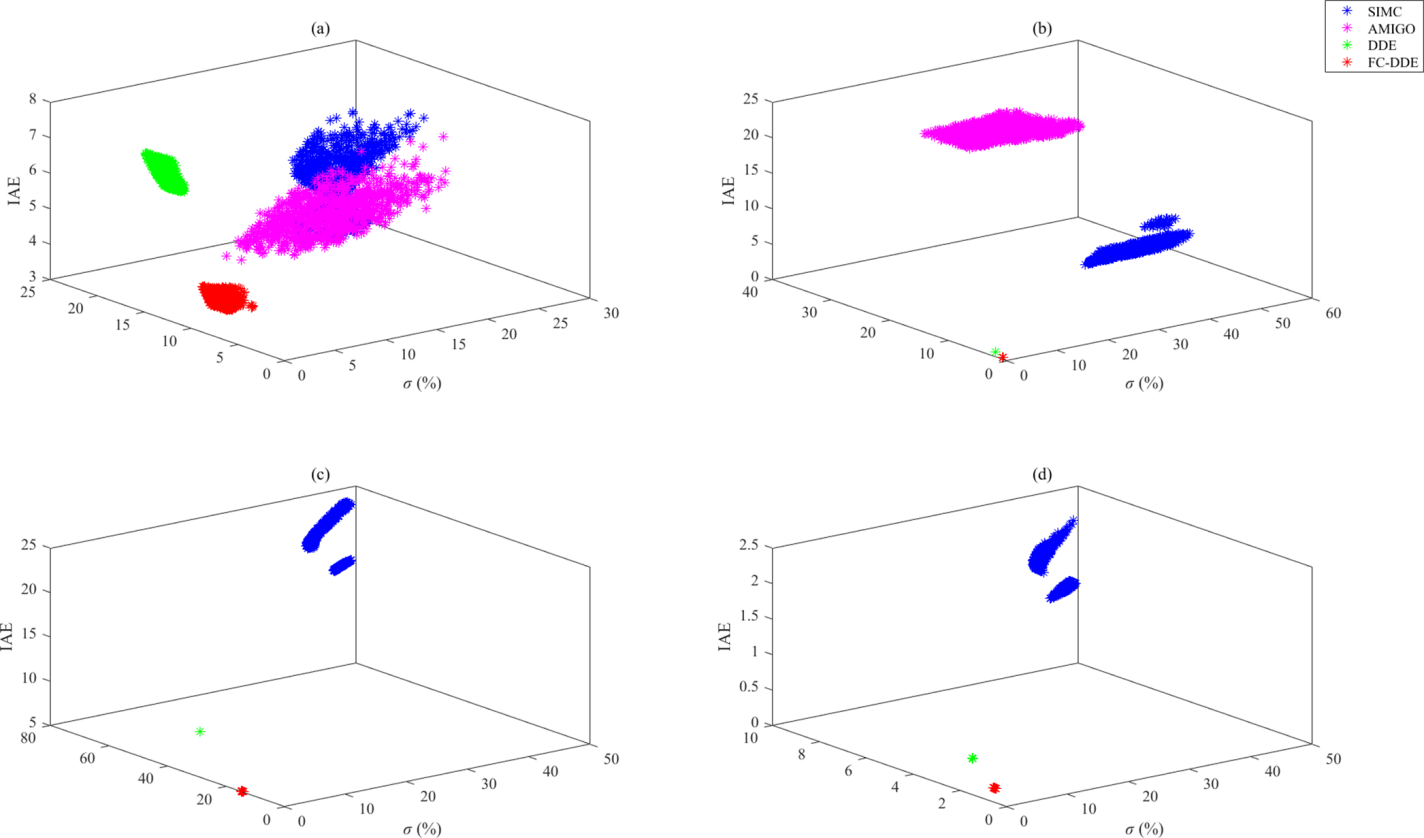
Results of Monte Carlo trials for each process: (a) *G*_p7_; (b) *G*_p8_; (c) *G*_p9_; and (d) *G*_p10_.

Uncertainties may exist in practical systems, so it is necessary to test the robustness of different controllers. Monte Carlo simulation is an effective method because it can intuitively indicate which controller has stronger robustness and better dynamic performance.^40^Figures 14 and 15 show results of 1000 times Monte Carlo trials for each process. Note that the coefficients of process models listed in Table 2 are perturbed within a range of ±20% and dynamic indices such as the overshoot, settling time, and the IAE are recorded during simulations. Besides, IAE refers to the sum of IAE_sp_ and IAE_ud_.

According to the results illustrated in Figures 14–15, we conclude the following:

(1) Compared with AMIGO PID and SIMC PID, scatter points of the FC-DDE PID are more intensive, which means that the proposed controller has stronger robustness.
(2) As for most of the processes listed in Table 2, DDE PID has stronger robustness than FC-DDE PID. However, its dynamic performance is worse than that of the proposed controller.

Based on all simulation results in this section, generally, FC-DDE PID can not only obtain satisfactory performance but also has strong robustness, which shows its potential for practical industrial systems.

## 6 Experimental Verification and Field Test

### 6.1 Experimental Tests on the Water Tank

Prior to industrial application, a laboratory experiment is necessary to confirm the feasibility of the method and the validity of the theoretical analysis and simulation results above.^41^ Therefore, the proposed controller is designed for the level control system of a water tank. In terms of practical systems, PID controllers are rarely used for the reason that the derivative action may lead to the self-oscillations of the control signal when measurement noise exists.^39^ As a result, PI controllers are usually applied to industrial process control. In Section 6, all controllers are designed based on PI controllers.

#### 6.1.1 Experimental Setup and Process Model

**Figure 16:**
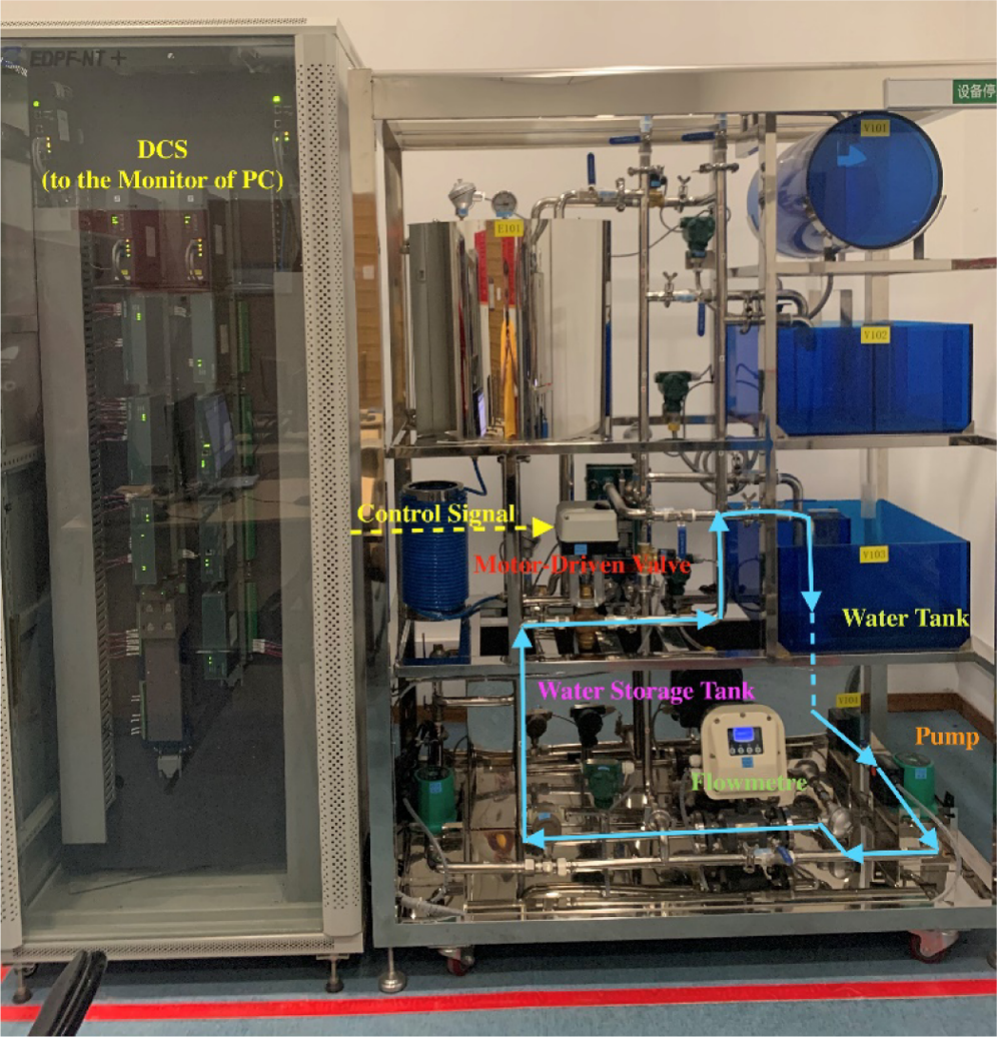
Experimental setup of the water tank.

Figure 16 shows the experimental setup of the water tank, which mainly includes the water tank, the storage tank, the motor-driven valve, and the DCS. Note that all controllers are implemented on the DCS whose sample time is 1 s.

**Figure 17:**
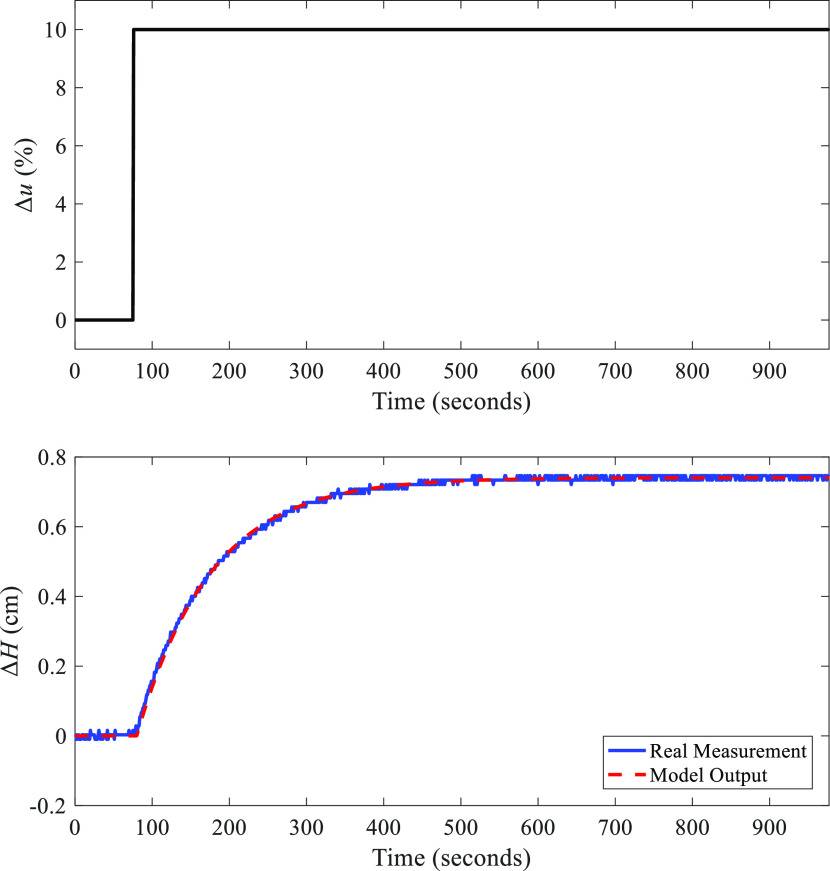
Result of the identification of the level system.

To design SIMC PI and AMIGO PI as the comparative controllers, the level system should be identified as an FOPDT process. Figure 17 shows the result of the identification.

In Figure 17, Δ*u* and Δ*H* are the changes of the valve opening and the water level, respectively. Based on Figure 17, the transfer function model of the water level can be depicted as
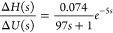
8

#### 6.1.2 Results and Discussion of Experiments

**Figure 18:**
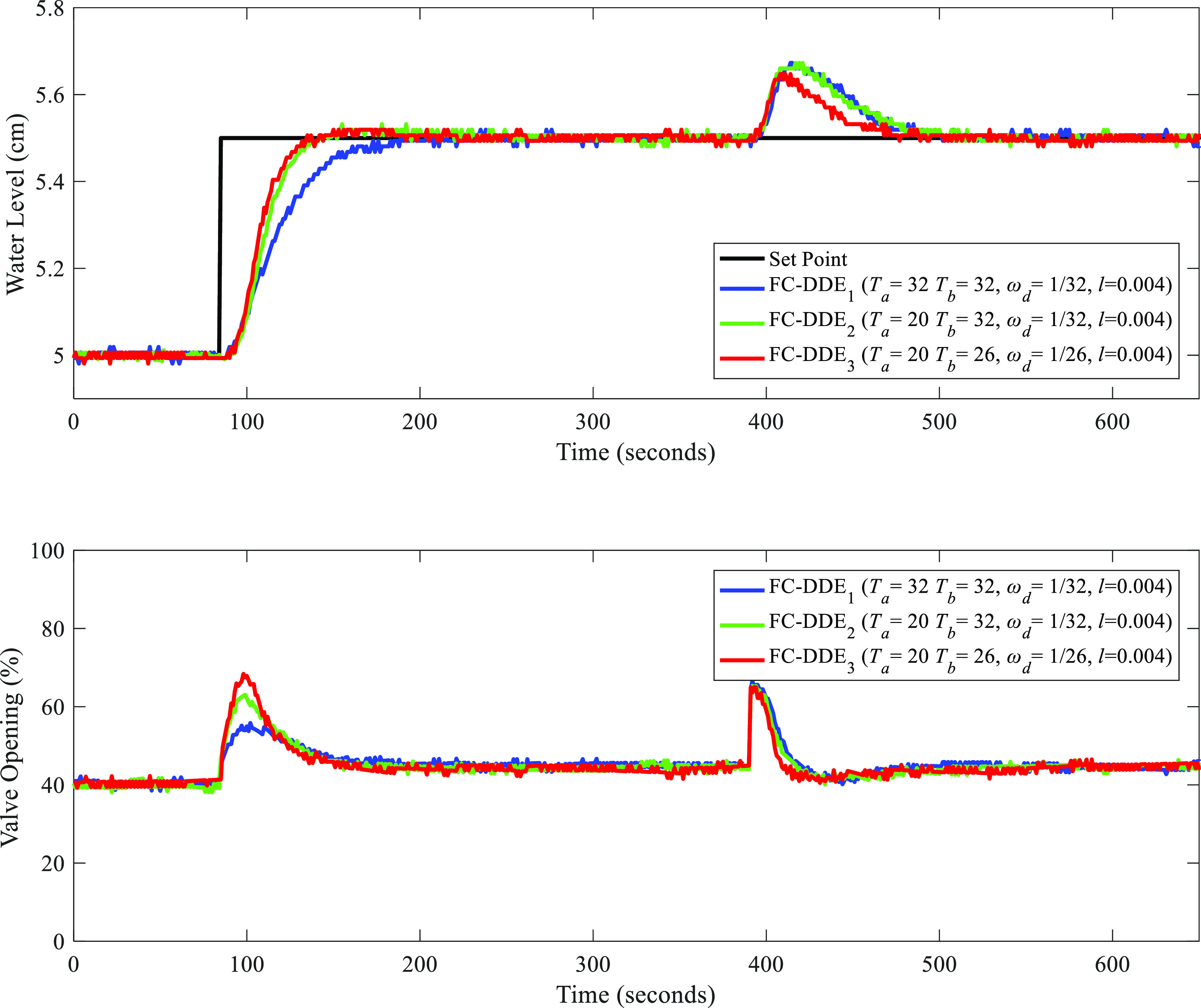
Experimental results of different FC-DDE PI controllers for the level system.

First, it is demonstrated by several experiments that FC-DDE PI can eliminate the conflict completely without using the process model. Three different FC-DDE PI controllers are designed for the water level control system. Figure 18 shows the experimental results of different FC-DDE PI controllers for the level control system. Note that the set point has a step change with the amplitude of 0.5 cm at 85 s, while an opening disturbance with the amplitude of 20% is added at 390 s.

The FC-DDE_1_ PI has the same parameters of PI controllers as FC-DDE_2_ PI, although the former one has a larger *T*_a_. As a result, their input–output responses are different and disturbance–output responses are almost coincident. Besides, FC-DDE_2_ PI has the same *T*_a_ as FC-DDE_3_ PI, while their parameters of PI controllers are different. Consequently, they achieve different disturbance rejection performances and the same reference tracking performance. Experimental results in Figure 18 demonstrate that the proposed controller can eliminate the conflict completely.

**Figure 19:**
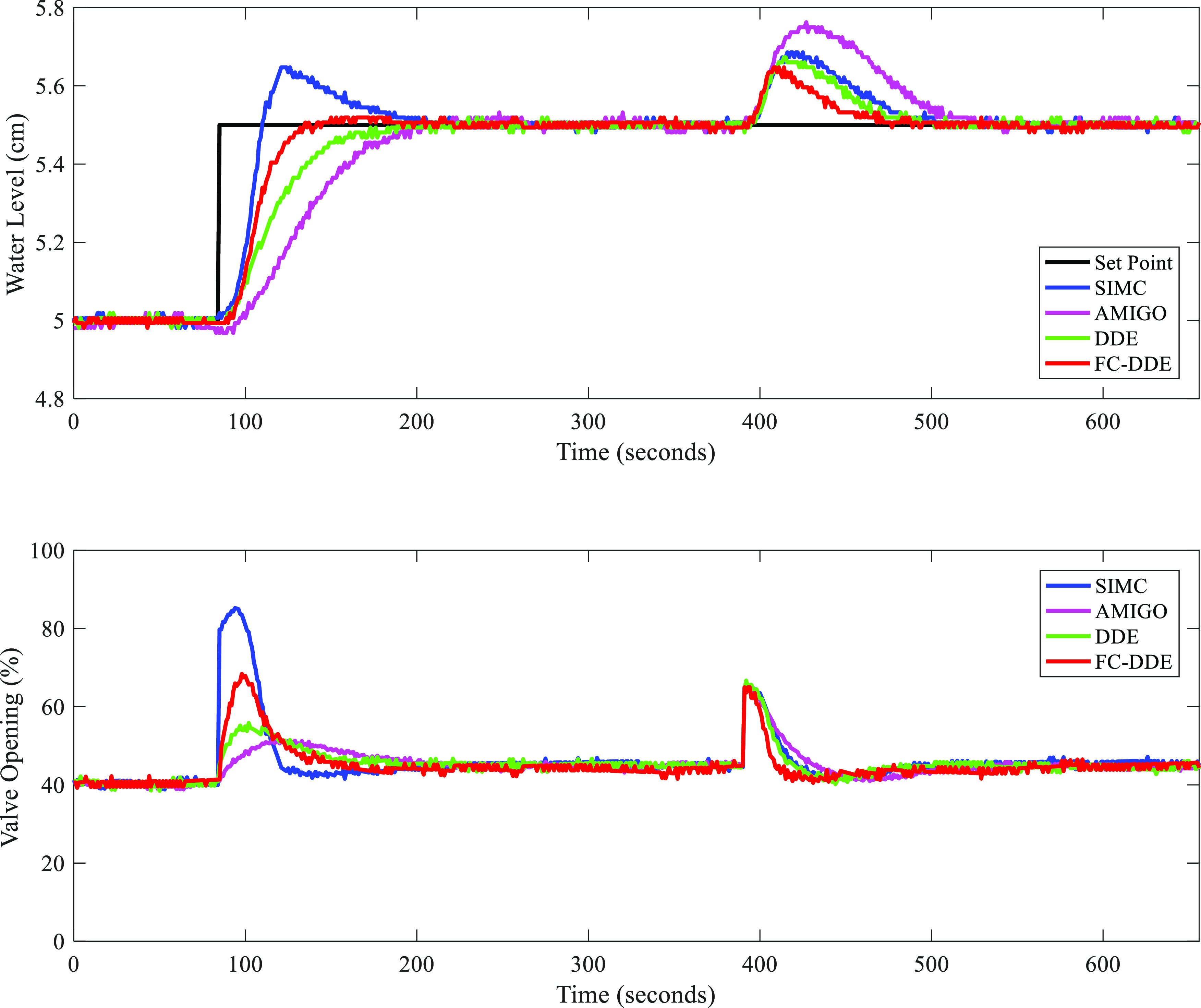
Experimental results of different controllers.

Second, comparative controllers, including SIMC PI, AMIGO PI, and DDE PID, are applied to the level control system. Table 5 lists the parameters of comparative controllers for the water tank. Based on the parameters listed in Table 5, Figure 19 shows the experimental results of different controllers. Note that “FC-DDE” refers to FC-DDE_3_ PI in Figure 18.

**Table 5:** Parameters of Comparative Controllers for the Level System

SIMC {*K*_p_, *T*_i_}	AMIGO {*K*_p_, *T*_i_, *b*}	DDE {*l*, *k*, ω_d_}
{131.08, 40}	{81.56, 41.45, 81.56}	{0.004, 5/16, 1/32}

From Figure 19, obviously, FC-DDE PI can achieve a faster tracking response than AMIGO PI and DDE PI and a smaller overshoot than SIMC PI, which shows its advantage in reference tracking. Moreover, when the disturbance is added, FC-DDE PI can eliminate the dynamic deviation with a faster speed than other comparative controllers, which demonstrates its superiority in disturbance rejection. To evaluate the control performance of different controllers for the level system quantitatively, Table 6 presents dynamic indices calculated based on experimental results, including the overshoot σ, the settling time *T*_s_, the IAEs, and the TV of the control signal. Note that IAE_sp_ is defined as the IAE of reference tracking, while IAE_ud_ is defined as that of disturbance rejection. Note that the settling time is calculated based on ±5% criterion.

**Table 6:** Dynamic Indices of Different Controllers for the Level Control of the Water Tank

controller	σ (%)	*T*_s_ (s)	IAE_sp_	IAE_ud_	TV
SIMC	29.49	104	16.7113	10.8525	489.22
AMIGO	6.41	237	29.1729	18.3909	322.15
DDE	3.85	94	19.5447	9.2371	523.53
FC-DDE	3.85	45	14.3524	6.6857	613.59

According to Table 6, compared with comparative controllers, the proposed controller has the smallest overshoot, the shortest settling time, and the smallest IAE_sp_ and IAE_ud_. However, FC-DDE PI has the largest TV, which means that the actuator was traded off to obtain better control performance. The experimental results demonstrate the effectiveness of the proposed FC-DDE PI.

Finally, the experimental results indicate that FC-DDE PI can eliminate the conflict completely without using the accurate process model and has advantages in both reference tracking and disturbance rejection.

### 6.2 Field Application to the High-Pressure Heater

Motivated by the encouraging results of simulations and the laboratory experiment, a field test is carried out as described in this subsection based on the proposed controller.

#### 6.2.1 Process Description

**Figure 20:**
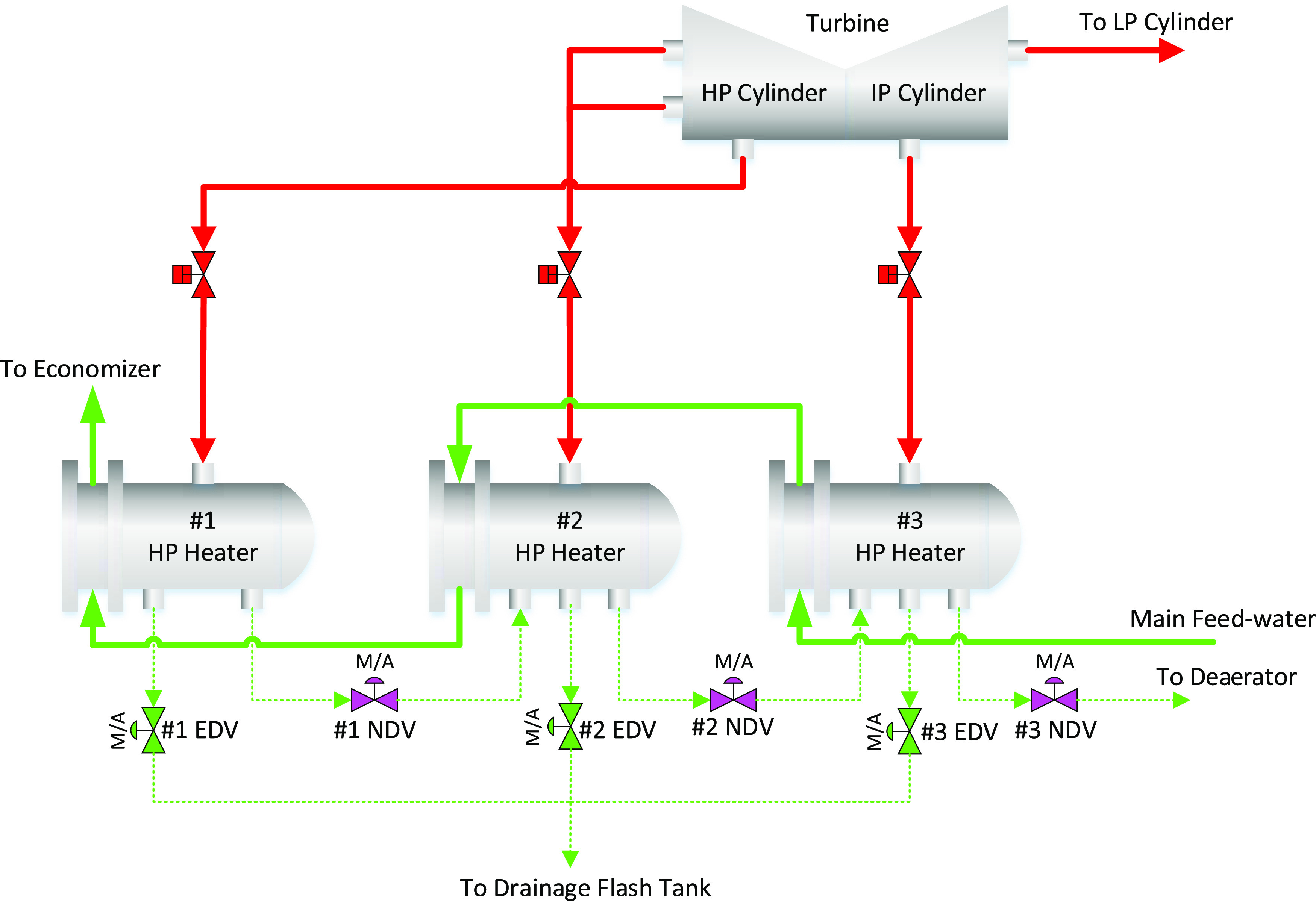
Schematic diagram of the HP steam extraction and drainage system (IP: intermediate pressure; LP: low pressure; EDV: emergency drainage valve; NDV: normal drainage valve; M/A: manual/auto).

FC-DDE PI is applied to a high-pressure (HP) heater of the HP steam extraction and drainage system in a 600 MW in-service air-cooling supercritical unit of a coal-fired power plant in Liaoning, China, whose schematic diagram is shown in Figure 20. The HP heater is an important component in the feedwater regenerative system of a power plant. It is used to heat the boiler feedwater with high-temperature steam, which is extracted from the turbine.^42^

The levels of HP heaters are of significance to the daily operation of a unit. A higher or lower level than the set point would deteriorate the thermal economy or even threaten the safety of the unit.^43^ Therefore, it is important to control the level of the HP heater at a desired value.

From Figure 20, it is obvious that the level of #2 HP heater is most difficult to control for the reason that it is influenced by levels of both #1 and #3 HP heaters. As a result, FC-DDE PI is designed to control the level of #2 HP heater to demonstrate its effectiveness. The manipulated variable and the controlled variable are the opening of #2 NDV and the level of #2 HP heater, respectively. There are three major sources of disturbances in this thermal process, including the opening of #2 EDV, the working fluid flux from #1 HP heater, and the steam flux from the HP cylinder. Compared with other sources of disturbances, the opening of #2 EDV has a more significant impact on the level. The control goals of the HP heater are listed as follows:

- The primary goal is to regulate the level of the HP heater as close to its set point as possible in the face of various disturbances.
- Reference tracking is another important goal, which is required when the unit is starting or stopping.

**Figure 21:**
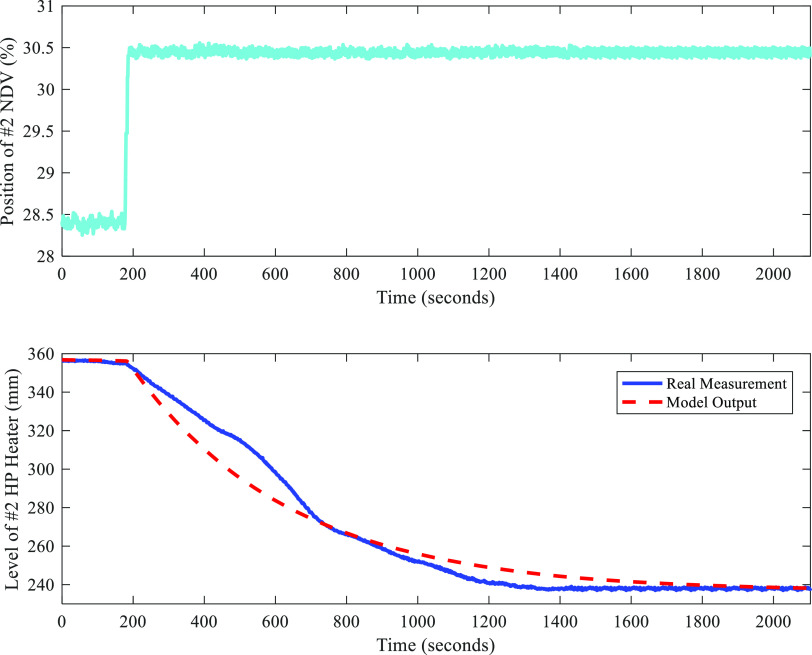
Comparison between the real measurement and the model output (date: Aug 31, 2021; time span: 11:00–11:36).

Based on an open-loop step response when the load was varying around 300 MW, the transfer function model from the position of #2 NDV to the level of #2 HP heater is identified as an FOPDT system depicted as
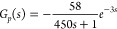
9The comparison between the real measurement and the model output is illustrated in Figure 21. It is obvious that the transfer function, depicted as eq 9, can describe the characteristics of the process.

#### 6.2.2 Results and Discussion of Field Tests

**Figure 22:**
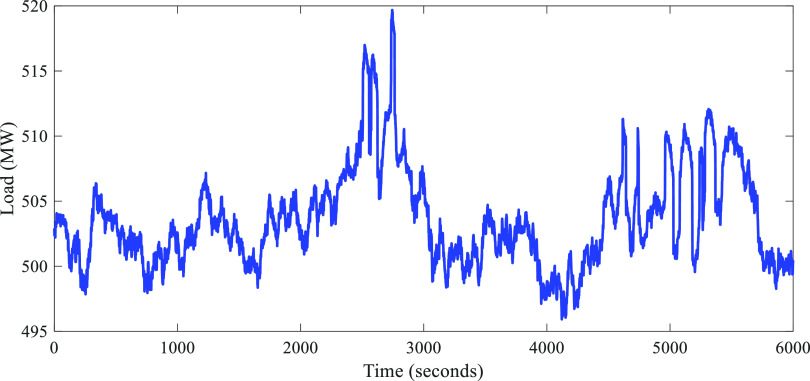
Variation of load (date: Sep 2, 2021; time span: 19:50–21:30).

All field tests were carried out from 19:50 to 21:30 on Sep 2, 2021. The variation of load during this period is presented in Figure 22.

From Figure 22, we can learn that the load varied within a range of 495 MW to 520 MW during field tests. However, the parameters of FC-DDE PI are tuned based on eq 9 on simulations, which means that the process model has changed when the tests are being carried out. Following results of field tests illustrate the strong robustness of the proposed controller.

**Figure 23:**
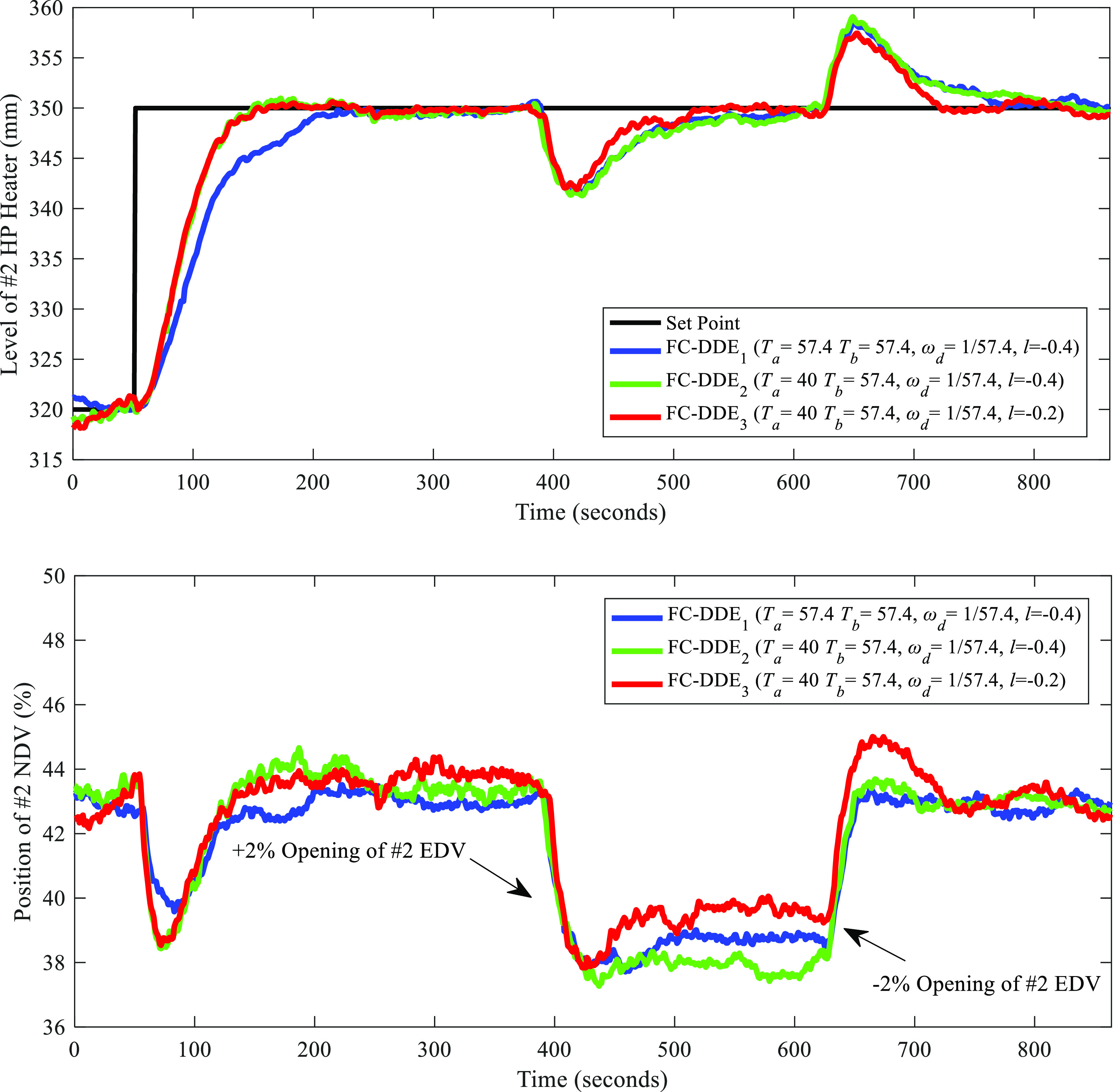
Field test results of different FC-DDE PI controllers (date: Sep 2, 2021; time span: FC-DDE_3_: 19:52:06–20:06:29; FC-DDE_2_: 20:24:06–20:38:29; FC-DDE_1_: 20:45:06−20:59:29).

Similar to Section 6.1, the ability of FC-DDE PI to completely eliminate the conflict between the input–output response and the disturbance–output response was validated first. For a fair comparison, the set point of the level was regulated in the same range and disturbances of the opening of #2 EDV with the amplitude of ±2% were added for different FC-DDE PI controllers. Figure 23 shows the field test results of different FC-DDE PI controllers for the level of #2 HP heater.

According to Figure 23, the following facts are obvious:

(1) Because of the different *T*_a_ and the same parameters of the PI controller, FC-DDE_1_ PI and FC-DDE_2_ PI obtain almost the same disturbance rejection performance and a different reference tracking performance.
(2) FC-DDE_2_ PI and FC-DDE_3_ PI achieve almost the same tracking responses and different disturbance rejection responses for the reason that they have different parameters of the PI controller and the same *T*_a_.

Therefore, the field test results illustrated in Figure 23 demonstrate that the proposed controller can achieve independent reference tracking performance and disturbance rejection performance.

**Figure 24:**
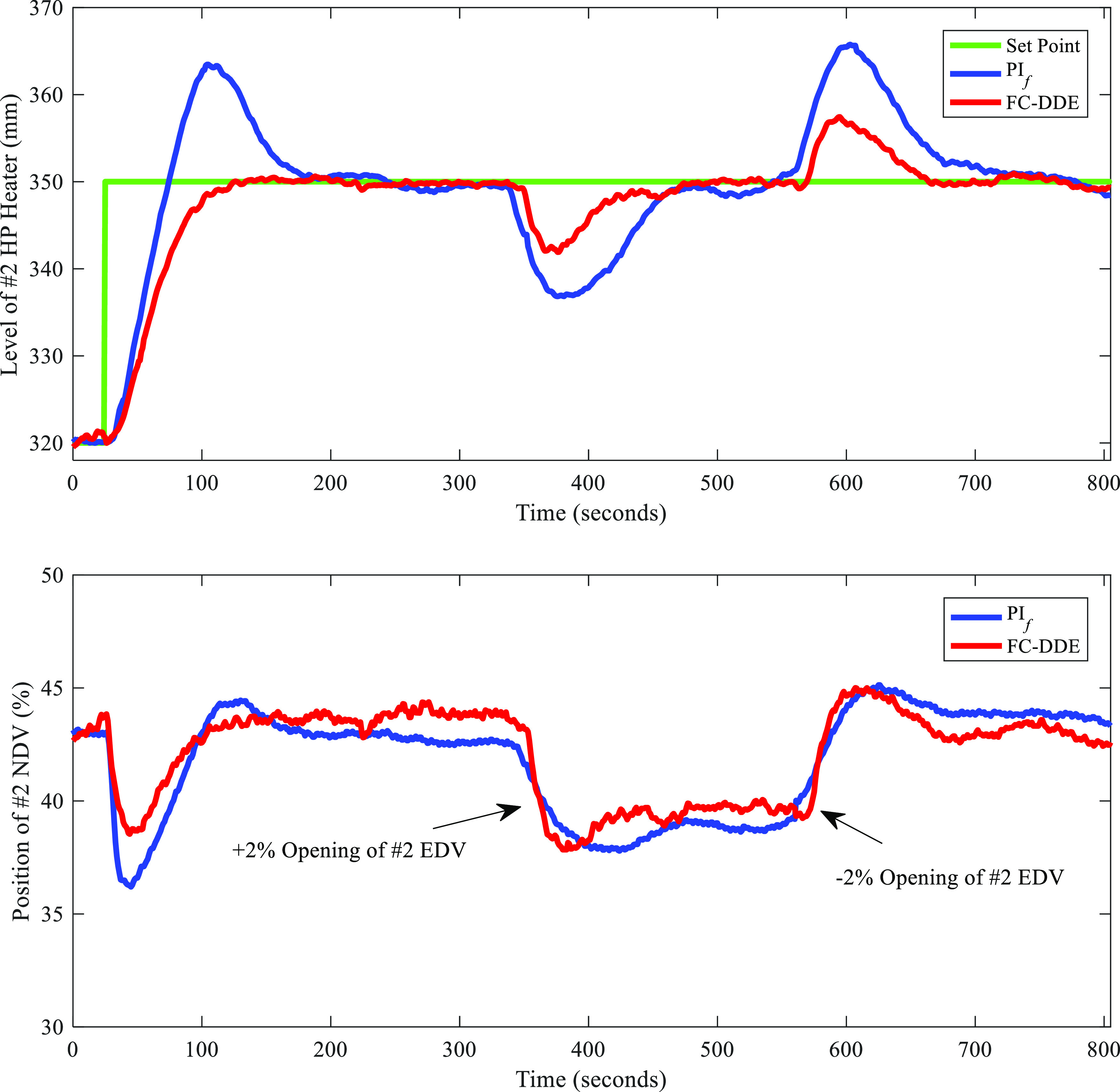
Comparison of control performance between FC-DDE PI and PI_f_ (date: Sep 2, 2021; time span of PI_f_: 21:05:48–21:19:14).

Then, the proposed FC-DDE PI was compared with the original PI controller, which was tuned by an experienced field engineer, and the parameters of the original PI controller are *k*_p_ = −2/9 and *k*_i_ = −1/297. Figure 24 illustrates the comparison of the control performance between the proposed FC-DDE PI and the original PI, which is denoted as “PI_f_”. Similarly, the set point of the level is regulated from 320 to 350 mm and disturbances of the opening of #2 EDV with the amplitude of ±2% were added for PI_f_ as well.

Note that FC-DDE PI refers to FC-DDE_3_ PI in Figure 23. Besides, for a fair comparison, the result of FC-DDE PI was processed by deleting static data to guarantee the same time span as PI_f_. From Figure 24, obviously, the original PI has a large overshoot and its dynamic deviations caused by disturbances are larger than those at FC-DDE PI. As a result, the control performance of the level of the HP heater is largely improved. To further demonstrate the merits of the proposed controller, Table 7 illustrates the performance indices of different controllers for the level control tests.

**Table 7:** Performance Indices of Different Controllers for the Level Control Tests

	reference tracking		disturbance rejection		TV
controller	*σ* (%)	*T*_*s*_ (s)	e^+^ (mm)	e^–^ (mm)	
PI_f_	45.0	143	15.6	13.2	55.92
FC-DDE	0.4	97	7.4	8.1	71.40

In Table 7, e^+^ and e^–^ denote the maximum positive deviation and the maximum negative deviation, respectively. According to Table 7, in terms of reference tracking, the proposed controller has a smaller overshoot and a shorter settling time than the original PI; as for disturbance rejection, FC-DDE PI can effectively eliminate the dynamic deviation. However, the TV of FC-DDE PI is larger than that of PI_f_, which means that the actuator was acting frequently to obtain better control performance.

The field tests confirm the merit of the proposed controller in terms of the TDOF structure nature. That is, the objectives of reference tracking and disturbance rejection can be tuned independently. The successful application to the level control of the HP heater indicates that the proposed controller has promising prospects in the control of coal-fired power plants.

## 7 Conclusions

In this paper, a novel quasi-model-free TDOF controller—FC-DDE PI/PID—is proposed to achieve independent reference tracking performance and disturbance rejection performance. According to the design, simulations, experiments, and field tests, some concluding remarks about the proposed controller are summarized as follows:

1. It can completely eliminate the conflict between the input–output response and the disturbance–output response and has no dependency on the accurate process model. However, the premise is that the output of DDE PI/PID should track the desired dynamic response precisely.
2. It is simple to implement on the DCS of the coal-fired power plant.
3. It has strong robustness so that uncertainties in thermal processes can be handled.

Our future work will focus the following areas:

1. The field application of FC-DDE PI/PID to other thermal processes of a coal-fired power plant.
2. The development of the auto-tuning toolbox of FC-DDE PI/PID.
3. FC-DDE PID design for infinite-dimensional systems.

## Figures and Tables

**Figure 25:**
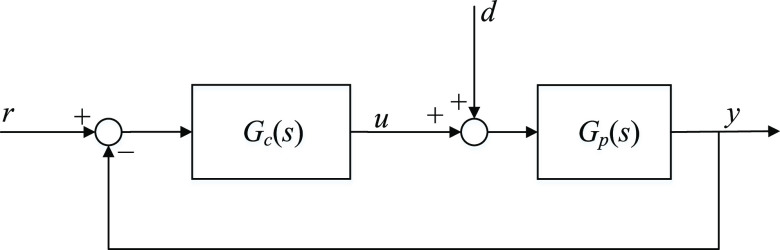
1-DOF control structure.

**Figure 26:**
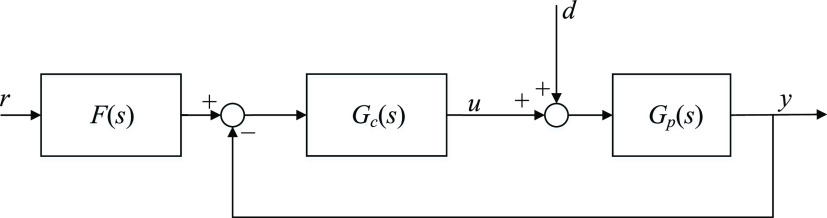
2-DOF control structure.

**Figure 27:**
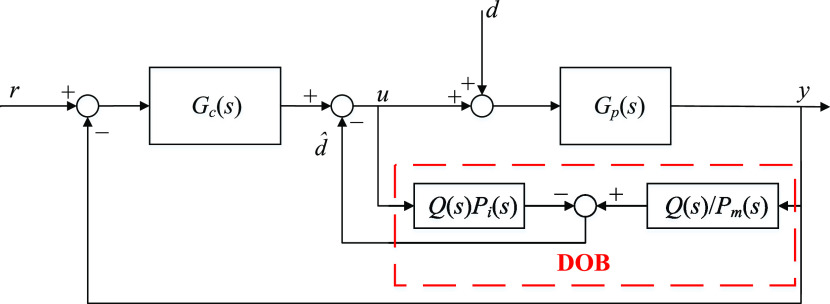
Structure of the DOB-based control system.

**Figure 28:**
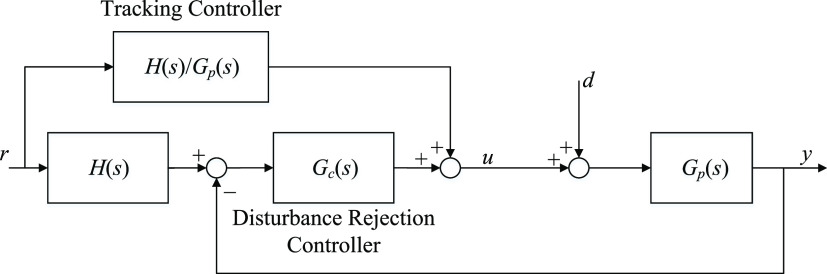
Structure of the CF control system.

## Appendix A. Analyses of Typical Control Systems

The 1-DOF control system, referring to the classical feedback control system, is illustrated in Figure 25.

In Figure 25, *G*_c_(*s*) represents that of the controller. Based on Figure 25, the transfer functions from *r* and *d* to *y* can be depicted as

10

where *R*(*s*), *D*(s), and *Y*(*s*) are the Laplace transformations of *r*, *d* and *y*, respectively. From eq 10, it is obvious that *G*_c_(*s*) determines both the input–output response and the disturbance–output response, which makes the two responses conflicting. Additionally, some of 1-DOF controllers are designed based on the accurate process model, such as MPC.

The structure of the classical 2-DOF control system is shown in Figure 26. It is the equivalent structure of the 2-DOF PI/PID controller^3^ and the linear active disturbance rejection controller (LADRC).^44−46^

In Figure 26, *F*(*s*) denotes the feedforward. According to Figure 26, the transfer functions from *r* and *d* to *y* can be depicted as

11

Based on eq 11, it is easy to learn that *F*(*s*) only determines the response from *r* to *y*, while *G*_c_(*s*) determines both the input–output response and the disturbance–output response. Therefore, the conflict is incompletely eliminated.

The structure of the traditional DOB-based control system is presented in Figure 27. The DOB is used to estimate and compensate for the external disturbances and uncertainties.

In Figure 27, *d̂* refers to the estimation of *d* and *Q*(*s*) is the filter of the DOB. In addition, *P*_m_(*s*) and *P*_i_(*s*) are depicted as the reversible part and the irreversible part of the plant, respectively, which means that

12

where *G̃*_p_(*s*) is considered as the estimated model of the plant. When the process model is matched (i.e., *G̃*_p_(*s*) = *G*_p_(*s*)), we have

13

Consequently, the response of the output can be depicted as

14

From eqs 13 and 14, it is evident that the disturbance–output response can only be modified by the DOB. However, the controller determines both the reference tracking and disturbance rejection. As a result, the conflict still exists. Moreover, according to eq 13, the DOB is designed based on the accurate process model.

The structure of CF control system is shown in Figure 28.^4^ It mainly consists of two parts: the tracking controller and the disturbance rejection controller.

In Figure 28, *H*(*s*) is the reference model of the closed-loop system. The transfer functions from *r* and *d* to *y* can be depicted as

15

From eq 15, obviously, the input–output response is determined by *H*(*s*), while the disturbance–output response is determined by *G*_c_(*s*), which means that the CF can eliminate the conflict. However, the tracking controller of CF is designed based on the accurate model of the plant, which is usually difficult to obtain for thermal processes.

## Appendix B. Principle of DDE PID

Suppose that the process can be depicted as a general system as follows

16

where *m*, *n*, τ, and *H* denote the order of the denominator, the relative degree, the delay time, and the high-frequency gain.^47^ Moreover, α_*i*_ (*i* = 1, 2, ···, *m* – *n* – 1) and β_*j*_ (*j* = 1, 2, ···, *m* – 1) are defined as coefficients of the numerator and the denominator, respectively. Note that α_*i*_ (*i* = 1, 2, ···, *m* – *n* – 1), β_*j*_ (*j* = 1, 2, ···, *m* – 1), and *H* are usually unknown.

The general process *G*_gp_(*s*) can be rewritten in the normalized state space form as

17

where *z*_*i*_ (*i* = 1, 2, ···, *n*) and *w*_*i*_ (*i* = 1, 2, ···, *m* – *n*) are defined as the state variables of the process. Besides, in eq 17, λ_*i*_ (*i* = 1, 2, ···, *n* – 1) and ζ_*i*_ (*i* = 1, 2, ···, *m* – *n* – 1) are unknown parameters.

Define an extended state *f* as

18

where *l* is defined as a parameter that has the same sign as *H*. Then *ż*_*n*_ in eq 17 can be rewritten as

19

If the process is regarded as a general second-order system, which means that *n* is equal to 2, its normalized state-space expressions can be derived as

20

Correspondingly, the desired dynamic equation is depicted as

21

Combined with eq 20, the control law should be designed as

22

However, *f* is uncertain, so it is estimated by the following disturbance observer algorithm

23

where *f̂* refers to the estimation of *f* and *k* denotes as the gain of the disturbance observer. Besides, ξ is defined as the intermediate variable. Therefore, when *f* → *f̂*, eq 22 can be rewritten as

24

According to eq 23, we have

25

Integrating both sides of eq 25, it is easy to learn that

26

Combined with eq 24, eq 26 can be written as

27

Due to the fact that *r* is a step change in practical processes, *r*^(1)^ is unbounded and can be set as zero.^48^ Moreover, define the tracking error as *e* = *r* – *z*_1;_ then we have *e*^(1)^ = *r*^(1)^ – *z*_1_^(1)^ = −*z*_2_. Therefore, eq 27 can be rewritten as

28

Moreover, if the process is considered a general first-order system, it is easy to derive the control law of DDE PI in the same way as

29
